# Integrated spatial-temporal feature alignment with graph convolutional and gated recurrent networks for traffic flow prediction

**DOI:** 10.1371/journal.pone.0337661

**Published:** 2026-04-28

**Authors:** Karimeh Ibrahim Ata, Mohd Khair Hassan, Syed Abdul Rahman Al-Haddad, Thamer. Alquthami, Ribhan Zafira Abdul Rahman, Sameer Alani, Md Azizul Hoque

**Affiliations:** 1 Artificial Intelligence and Sensing Technologies (AIST) Research Center, University of Tabuk, Tabuk, Saudi Arabia; 2 Department of Computer Engineering, Faculty of Computers and Information Technology, University of Tabuk, Tabuk, Saudi Arabia; 3 Department of Electrical and Electronic Engineering, Faculty of Engineering, University Putra Malysia, UPM Serdang, Selangor Darul Ehsan, Malaysia; 4 Department of Computer and Communication Systems Engineering, Faculty of Engineering, University Putra Malysia, UPM Serdang, Selangor Darul Ehsan, Malaysia; 5 Department of Electrical and Computer Engineering, King Abdulaziz University, Jeddah, Saudi Arabia; 6 Electronic Computer Center, University of Anbar, Iraq; National University of Defense Technology, CHINA

## Abstract

Accurate traffic flow prediction is essential for Intelligent Transportation Systems (ITS), yet capturing the complex spatiotemporal relationships within traffic data remains challenging due to dynamic traffic patterns and the non-Euclidean structure of road networks. Existing models struggle to adapt in real time, limiting their prediction accuracy and reliability. This study introduces the Spatiotemporal Feature Alignment with Graph Convolutional and Gated Recurrent Unit (STF-GGRU) model to address these limitations. By integrating a novel Integrated Spatiotemporal Feature Alignment (ISTFA) module, which combines Dynamic K-Nearest Neighbor (D-KNN) and Centered Kernel Alignment (CKA), the model dynamically captures critical spatial and temporal interactions. The STF-GGRU model achieves superior prediction accuracy, with RMSE values of 27.18 and 11.1 on the PeMSD4 and PeMSD8 datasets, outperforming traditional methods such as ARIMA, GRU, LSTM, and advanced neural models. These results demonstrate STF-GGRU’s potential for robust, real-time traffic predictions, marking a significant advancement in ITS capabilities.

## 1. Introduction

With rapid urbanization and the explosive growth of transportation networks worldwide, urban traffic congestion has emerged as one of the most pressing societal challenges. Traffic congestion not only causes significant economic losses, estimated at hundreds of billions of dollars annually, but also contributes to increased greenhouse gas emissions, environmental degradation, and reduced quality of life for urban residents. Accurate and timely traffic flow prediction is a critical component of Intelligent Transportation Systems (ITS). It empowers city planners and traffic operators to implement proactive congestion management, dynamic route guidance, and adaptive traffic signal control [1].

However, traffic flow prediction remains inherently difficult because of its highly nonlinear, dynamic, and spatiotemporally correlated nature. Traffic conditions are influenced not only by local patterns but also by distant upstream or downstream interactions, unpredictable events (e.g., accidents, public gatherings), and external factors (e.g., weather conditions, roadwork). These complex dependencies require sophisticated modeling techniques that can capture both spatial correlations across the road network and temporal evolution over time [2]. Traditional statistical approaches, such as Historical Average (HA), Autoregressive Integrated Moving Average (ARIMA), and Vector Autoregression (VAR), offer simplicity and computational efficiency. However, they are built on restrictive assumptions of linearity and stationarity [3–5]. Consequently, they often fail to adapt to abrupt changes and nonlinear interactions present in real-world traffic systems [6]. Machine learning methods such as K-Nearest Neighbor (KNN) and Support Vector Regression (SVR) introduced more flexibility by learning from historical patterns without requiring explicit model formulations. Nonetheless, they rely heavily on manually engineered features and lack the capacity to model deeper, hierarchical spatiotemporal relationships [6].

Recent advances in intelligent transportation research emphasize the importance of optimizing sensation, perception, and data-driven decision-making in complex traffic environments. For example, recent studies have investigated the optimal deployment of roadside sensing systems, such as LiDAR-based cooperative perception, to improve vehicle detection accuracy and traffic awareness in dense and dynamic traffic conditions [7]. These works demonstrate that intelligent sensing configuration and adaptive modeling are essential for improving traffic system performance, reinforcing the need for predictive models that can dynamically adapt to evolving spatiotemporal patterns.

The rise of deep learning has revolutionized traffic prediction by enabling automatic hierarchical feature extraction. Temporal modeling methods, including Recurrent Neural Networks (RNNs) [8], Long Short-Term Memory (LSTM) networks [9], and Gated Recurrent Units (GRUs) [10], have demonstrated strong capabilities in capturing sequential dependencies and long-term temporal patterns. Yet their focus is primarily on temporal dynamics, often overlooking intricate spatial relationships inherent in urban road networks [8].

To address spatial dependencies, Convolutional Neural Networks (CNNs) have been used to capture local spatial features. However, they are limited by their reliance on grid-like structures, which do not accurately represent the non-Euclidean topology of real-world road networks [9]. Graph Convolutional Networks (GCNs) overcome this limitation by effectively modeling traffic data as graphs, capturing complex spatial correlations across irregular and dynamic urban networks [10]. Hybrid architectures, such as Temporal Graph Convolutional Networks (T-GCN) and Diffusion Convolutional Recurrent Neural Networks (DCRNN), integrate both spatial and temporal features by combining GCNs and recurrent structures [11], [12]. Despite this progress, most models still depend on static or predefined graph structures. This limits their adaptability in dynamic scenarios such as accidents, unexpected events, or temporary road closures [13]. Recent studies have explored dynamic graph structures and attention mechanisms to improve flexibility and accuracy. Dynamic spatiotemporal GCNs update graph connectivity over time [14], while attention-based networks selectively emphasize critical spatiotemporal patterns to improve learning performance [15]. Furthermore, Generative Adversarial Networks (GANs) have been employed to augment data and improve model robustness against noise and sparsity [16]. Although these methods represent significant steps forward, they typically fail to explicitly integrate feature-based similarities across sensors. They also often do not provide a unified, adaptive framework capable of simultaneously modeling both spatial proximity and feature relationships in real time [17]. To address these challenges, we propose a novel and unified traffic flow prediction framework named STF-GGRU (Spatiotemporal Feature-aligned Graph Gated Recurrent Unit). The core innovation lies in the Integrated Spatiotemporal Feature Alignment (ISTFA) module, which explicitly integrates both spatial proximity and feature-based similarity using a combination of Dynamic K-Nearest Neighbor (D-KNN) and Centered Kernel Alignment (CKA). Unlike previous approaches that rely on static adjacency matrices or focus solely on attention mechanisms, ISTFA provides a dynamic and unified feature representation that evolves adaptively as traffic conditions change.

The primary contributions of this work are summarized as follows:

1. A novel Integrated Spatiotemporal Feature Alignment (ISTFA) module: We propose a unique module that dynamically integrates spatial proximity and feature-based similarities using D-KNN and CKA, providing a more comprehensive and flexible graph representation. To our knowledge, this is the first attempt to combine these techniques for traffic flow prediction.
2. A hybrid STF-GGRU architecture: We develop a hybrid model that combines Graph Convolutional Networks (GCNs) with Gated Recurrent Units (GRUs) to jointly capture complex spatial correlations and temporal dependencies, improving predictive accuracy and robustness.
3. Extensive validation on large-scale real-world datasets: We rigorously evaluate STF-GGRU on two widely used urban traffic datasets, METR-LA and PEMS-BAY. Experimental results demonstrate that STF-GGRU consistently outperforms state-of-the-art baseline models across multiple horizons and metrics, confirming its practical effectiveness.
4. Comprehensive scalability analysis: We conduct additional experiments to analyze the scalability of STF-GGRU under different network sizes and sensor densities. The results indicate that STF-GGRU maintains stable performance and computational efficiency, highlighting its potential for real-world deployment in large-scale urban transportation systems.
5. Enhanced interpretability and deployment readiness: By integrating feature alignment and adaptive graph construction, STF-GGRU offers better interpretability of traffic relationships and is designed to support integration with distributed or edge computing environments, paving the way for intelligent and responsive traffic management applications.

The remainder of this paper is organized as follows. Section 2 presents a comprehensive review. Section 3 details the proposed STF-GGRU model and its key modules. Section 4 describes experimental setup. section 5 presents the dataset. evaluation metrics is presented in section 6. The results of the article is presented in sction 7.. Section 8 discussesthe ablation of the styudy.,. Finally, Section 9 concludes the paper and presents the future directions

## 2. Related work

Accurate traffic flow prediction is a cornerstone of intelligent transportation systems (ITS), directly impacting congestion mitigation, urban mobility planning, and safety. Classical statistical methods, such as Historical Average (HA), Autoregressive Integrated Moving Average (ARIMA), and Vector Autoregression (VAR), have been widely used due to their simplicity and interpretability [18,19]. However, these methods rely on assumptions of linearity and stationarity and often fail to capture abrupt and nonlinear variations characteristic of real-world urban traffic patterns, especially under incident scenarios or abnormal conditions. Machine learning techniques such as K-Nearest Neighbor (KNN) and Support Vector Regression (SVR) provide better adaptability by learning from data without explicit parametric assumptions [20,21]. Nonetheless, these methods depend heavily on manually engineered features and lack the capacity to capture hierarchical, nonlinear relationships, making them unsuitable for large-scale dynamic environments.

The advent of deep learning has fundamentally transformed traffic prediction. Recurrent Neural Networks (RNNs) and Long Short-Term Memory (LSTM) networks effectively model temporal dependencies by learning from sequential patterns [2,22]. However, LSTMs are resource-intensive and prone to overfitting, particularly when trained on sparse or noisy datasets [23]. Gated Recurrent Units (GRUs) simplify the gating structure and retain comparable performance while reducing computational demands [24]. Despite this, RNN-based models often focus solely on temporal dependencies and struggle to fully capture complex spatial relationships present in large sensor networks.

To address spatial dependencies, Convolutional Neural Networks (CNNs) have been applied but are limited by their requirement for grid-structured data, which does not fit the non-Euclidean nature of road networks [25]. Graph Convolutional Networks (GCNs) overcome this limitation by modeling traffic as a graph structure, effectively capturing irregular topologies and non-local spatial correlations [26]. Yet, most GCN-based models utilize static adjacency matrices derived from historical connectivity or road maps, thus failing to adapt to dynamic and evolving traffic conditions.

Hybrid models that combine spatial and temporal learning have shown promising improvements. The Temporal Graph Convolutional Network (T-GCN) integrates GCNs with GRUs to jointly learn spatial and temporal features [27], while the Diffusion Convolutional Recurrent Neural Network (DCRNN) models traffic as a diffusion process over a graph, effectively capturing propagation dynamics [28]. These approaches mark significant advances but mainly focus on spatial adaptivity, often overlooking detailed feature-level similarity between sensors. A notable contribution by Chen et al. [29]proposed the TFM-GCAM model, which integrates attention mechanisms with graph convolution and constructs a traffic flow matrix grounded in traffic flow theory. This framework successfully captures dynamic node relationships and fuses spatiotemporal features, outperforming more conventional hybrid architectures developed at the Beijing Institute of Technology. However, similar to other attention-based approaches, TFM-GCAM relies predominantly on learned attention weights and does not explicitly integrate dynamic feature similarity matrices across sensors, potentially limiting its interpretability and ability to generalize across diverse sensor types. Yan et al. [30] proposed a vehicle path planning and prediction algorithm utilizes attention mechanisms at complex intersections. Their approach significantly improved collaborative decision-making and safety at intersections but focused primarily on short-term vehicle trajectory prediction rather than holistic network-level flow forecasting. This limits its direct applicability to large-scale traffic flow prediction tasks.

Furthermore, Ahmad Ali et al. [31] introduced a data aggregation-based approach to exploit dynamic spatiotemporal correlations for citywide crowd flow prediction within fog computing environments. Their method emphasized scalability and edge-computing capabilities, addressing resource constraints in distributed systems. Despite its effectiveness in decentralized settings, the approach primarily targets crowd dynamics rather than continuous vehicle traffic flow and lacks fine-grained feature similarity integration, which is crucial for high-fidelity predictions in vehicular networks. Parallel to these graph-based advancements, Generative Adversarial Networks (GANs) have been increasingly employed to generate synthetic traffic data, augment sparse datasets, and enhance prediction robustness [32]. The systematic review highlighted GANs’ potential in improving data diversity and addressing missing data issues. However, GAN-based approaches primarily focus on data generation and augmentation rather than directly modeling spatiotemporal dependencies for predictive purposes. Attention-based models, such as the Attention-based Periodic-Temporal Neural Network (APTN) [33], further advance the field by enabling selective focus on relevant temporal segments and periodic patterns. These models have demonstrated superior performance, especially in highly dynamic and periodic traffic scenarios. Nevertheless, attention mechanisms alone cannot fully substitute for robust, dynamically integrated spatial and feature similarity modeling, which remains essential for network-level prediction accuracy and interpretability. Another line of research has focused on dynamic spatiotemporal graph construction, which aims to update graph structures as traffic conditions evolve. Two representative studies that fall within this category are DHSTNet and GSTRGCT. DHSTNet, introduced by Ali et al. [34], adaptively updates spatiotemporal graph structures and captures multi-scale spatial correlations. Although DHSTNet improves spatial adaptivity, it relies solely on structural correlations and does not incorporate feature-level similarity. As a result, it cannot combine spatial and feature information, and it offers limited interpretability and responsiveness to abrupt traffic changes.

GSTRGCT, proposed by Xiong et al. [35], employs tensor decomposition and autocorrelation-driven attention to model non-stationary spatiotemporal patterns. This approach captures complex dynamics but is computationally intensive and difficult to interpret. It also lacks a kernel-based similarity mechanism, and its transformer architecture reacts slowly to rapid fluctuations in traffic. Furthermore, GSTRGCT does not integrate spatial, temporal, and feature similarity within a unified framework. Table 1 provides a comparative overview of DHSTNet, GSTRGCT, ISTFA, and STF-GGRU.

**Table 1:** Comparison of dynamic graph methods and proposed models.

Capability / Property	DHSTNet	GSTRGCT	ISTFA (Module)	STF-GGRU (Full Model)
**Real-time dynamic graphs**	✔	✔	✔	✔
**Kernel-based similarity**			✔	✔
**Spatial + feature similarity fusion**			✔	✔
**Abrupt-change adaptation**	Partial	Partial	✔	✔
**Interpretable similarity scores**			✔	✔
**Long-range temporal modelling**	✔	✔		✔
**Heterogeneous sensor handling**			✔	✔
**Real-time efficiency**	✔		✔	✔
**Unified spatiotemporal–feature fusion**		Partial	Partial	✔
**High interpretability**	Partial		✔	✔
**Addresses key limitations**			Partial	✔

Despite significant progress in traffic prediction, many existing models still face important limitations. Many approaches rely on static or partially dynamic graph structures and do not include feature-based sensor similarity. This reduces their flexibility when traffic patterns shift suddenly. It also limits their ability to capture detailed relationships between sensors in real time. Although several studies have explored dynamic graph construction, most of these methods depend mainly on structural updates. Many also use isolated attention mechanisms that do not create deeper semantic alignment between sensor features. Table 2 provides a clear summary of the related traffic prediction studies, outlining their main approaches, key features, and observed limitations.

**Table 2:** Summary of the related works.

Model / Approach	Key Features	Advantages	Limitations
**HA, ARIMA, VAR**	Classical statistical models; linear, stationary assumptions	Simple, interpretable	Fail to capture nonlinear and dynamic patterns
**KNN, SVR**	Data-driven, manual feature engineering	Flexible, no strong parametric assumptions	Heavy feature engineering, poor scalability
**RNN, LSTM**	Temporal sequence modeling	Capture long-term dependencies	High complexity, prone to overfitting
**GRU**	Simplified recurrent gating	Less complex than LSTM, efficient	Limited spatial modeling, focuses on temporal only
**CNN**	Grid-based spatial feature extraction	Good for local spatial patterns	Cannot handle non-Euclidean networks
**GCN**	Graph-based spatial modeling	Effective for irregular network structures	Mostly static graphs, poor adaptability
**T-GCN, DCRNN**	Hybrid GCN-RNN or diffusion-based graphs	Joint spatiotemporal modeling	Use static/predefined graph structures
**DHSTNet**	Dynamic spatiotemporal GCN, multi-scale graphs	Adaptive to evolving patterns	Lacks explicit feature similarity integration
**GSTRGCT**	Graph transformers, tensor decomposition, autocorrelation	Model non-stationary dependencies	High complexity, limited real-time performance
**Data aggregation dynamic GCN**	Fog computing, dynamic spatiotemporal graphs	Scalable, edge-computing compatible	Targets crowd flows, lacks fine-grained feature fusion
**Attention-based neural network**	Dynamic attention to spatiotemporal correlations	Highlights critical patterns	No explicit kernel-based feature similarity
**Attention vehicle prediction**	Attention-based path prediction at intersections	Enhances safety, local decision-making	Not designed for network-wide flow prediction
**APTN**	Periodic attention for temporal patterns	Good for periodic modeling	Limited spatial feature integration
**GAN-based**	Generative data augmentation and robustness	Handles data sparsity, improves generalization	Focus on data generation, not direct prediction
**STF-GGRU (Proposed)**	Dynamic KNN, CKA, integrated GCN-GRU	Unified, adaptive, captures spatial & feature similarity	Robust, real-time adaptable, balanced complexity

To address these gaps, recent research has introduced techniques that incorporate richer feature information into dynamic graph learning. The ISTFA module contributes to the development of dynamic graph construction with explicit feature similarity. It does this by combining Dynamic K-Nearest Neighbour (D-KNN) with Centred Kernel Alignment (CKA). This combination quantifies feature-level similarity in a clear and interpretable way. It also supports fast and adaptive updates to the graph structure. As a result, the representation can evolve with changes in traffic conditions and sensor behaviours.

When used within a broader GCN-GRU architecture such as STF-GGRU, ISTFA provides a unified framework that brings together spatial proximity, temporal dependencies, and feature similarity. This integration improves the model’s ability to capture complex, context-dependent traffic patterns. It also maintains computational efficiency that is suitable for real-time prediction.

## 3. Materials and methods

### 3.1. Problem definition

Traffic congestion is a critical issue in modern cities, directly affecting travel time, fuel consumption, and air pollution. To manage traffic effectively, it is essential to accurately predict future traffic conditions. Traffic flow prediction aims to forecast the number of vehicles, their speed, and occupancy at specific locations and times, based on previously observed traffic patterns. Formally, the problem can be defined as forecasting future traffic flow using past traffic data collected from road sensors. This data is represented as a multivariate time series:


$${𝐗}_{t}={\left[x_{\text{1},t},x_{\text{2},t},\ldots ,x_{N,t}\right]}^{⊤},$$ (1)

where N=307 for PeMSD4 and N=170N for PeMSD8.

Spatial dependencies among sensors are modeled as a graph:


$$G=(\nu ,\varepsilon ,𝐀(t)),𝐀(t)\in R^{N\times N}$$ (2)

where A(t) is the time-varying adjacency matrix that captures relationships between sensors, including both sparse and direct connections.

The goal is to predict future traffic flow ${\hat{\text{X}}}_{t+k}$ at time t+k using data from the past Ttime steps:


$${\hat{\text{X}}}_{t+k}=f\left({\text{X}}_{t-T+\text{1}:t},{\text{A}}_{t-T+\text{1}:t}\right),$$ (3)

where f is the prediction function, and the objective is to minimize the error between the predicted and actual traffic flow:


$$L=\frac{\text{1}}{N}\sum_{i=\text{1}}^{N}\;{\left({\hat{x}}_{i,t+k}-x_{i,t+k}\right)}^{\text{2}}.$$ (4)

where N is the total number of sensors, ${\hat{x}}_{i,t+k}$ is the predicted traffic flow of sensor i at time t+k, and $x_{i,t+k}$ is the actual observed traffic flow of sensor i at the same time. The loss function L represents the Mean Squared Error (MSE), which measures the average squared difference between the predicted and actual values. The objective of the model is to minimize this error to improve the accuracy of traffic flow forecasting. To address this problem, a comprehensive framework is required to integrate key components: an optimized spatial kernel to capture localized spatial dependencies, an optimized feature kernel to model global temporal patterns, and adjacency matrices to represent raw and bidirectional spatial relationships. Hence, ISTFA is proposed as in formula 20.

### 3.2. Overview of the proposed model

**Fig 1:**
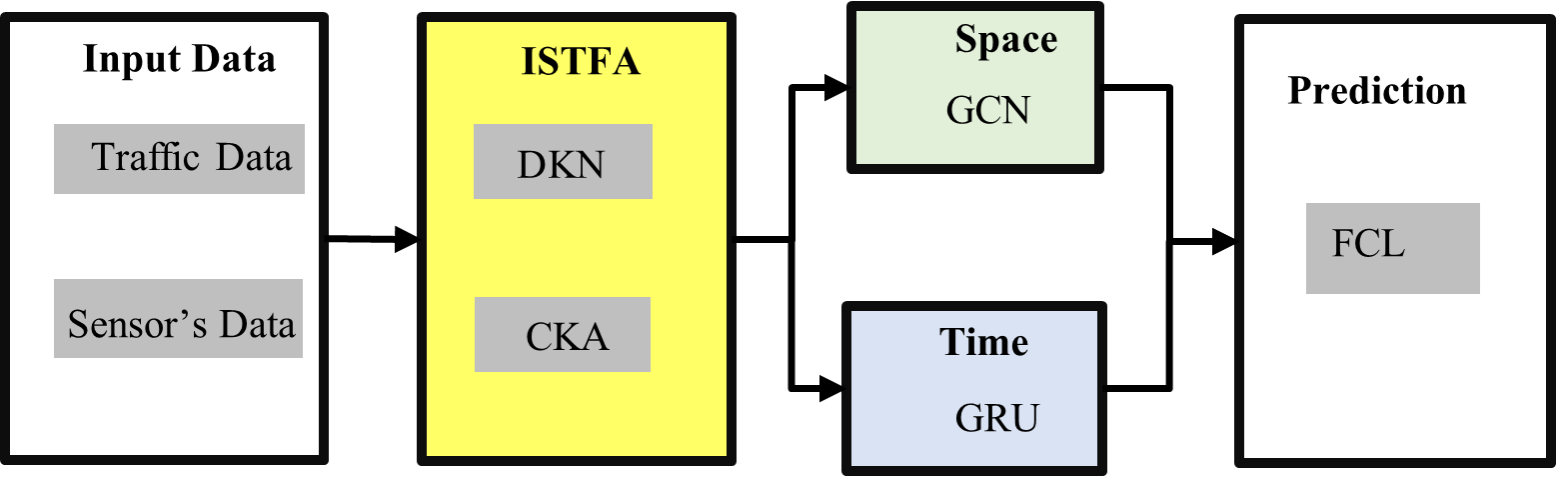
The Proposed STF-GGRU.

The STF-GGRU model presented in this study is structured into three key components: (1) the Feature Extraction Module, (2) the Deep Learning Modules encompassing both spatial and temporal learning, and (3) the Prediction Module, as illustrated in Fig 1. The STF-GGRU begins with collecting and preparing traffic data from 307 sensors, each recording speed, occupancy, and flow measurements every 5 minutes over a two-month period. To make sense of this high-dimensional data, the measurements are aggregated into daily, hourly, and weekly patterns using statistical methods such as mean, standard deviation, and min-max scaling. This aggregation reduces dimensionality and reveals meaningful patterns in the data, providing a clearer picture of traffic behaviour for each sensor over time.

Once the data is prepared, it enters the ISTFA module. Here, the D-KNN method identifies relevant neighbors for each sensor in two ways: spatially and by feature similarity. The spatial neighbours are identified based on the geographic distance between sensors, ensuring that nearby sensors are considered together. In contrast, the feature-based neighbors are determined by comparing aggregated traffic patterns, such as daily or weekly similarities, to find sensors with similar traffic behaviour. This two-fold neighbors selection provides a rich understanding of both geographic proximity and behaviourally similar sensors. To refine this selection, the CKA technique is then applied to align the spatial and feature-based neighbor matrices. CKA maximizes the congruence between these matrices, retaining only those neighbors that share both spatial and feature-based similarities. The result is a merged, comprehensive neighbor matrix that captures the most relevant spatiotemporal relationships for each sensor, which are essential for accurate traffic prediction. With the refined neighbor matrix as input, the model splits the data into spatial and temporal pathways. The GCN layer processes the spatial relationships, learning patterns and dependencies in the sensor network structure. Simultaneously, the GRU layer handles the temporal aspect, capturing sequential dependencies over time to understand how traffic patterns evolve. Finally, the outputs from the spatial GCN and temporal GRU pathways converge in the FCL layers, which synthesize these insights to produce the final traffic flow predictions. This layered approach, moving from raw sensor data through spatiotemporal alignment to predictive modelling, enables the proposed model to deliver precise and contextually relevant traffic forecasts.

### 3.3. Input module

**Fig 2:**
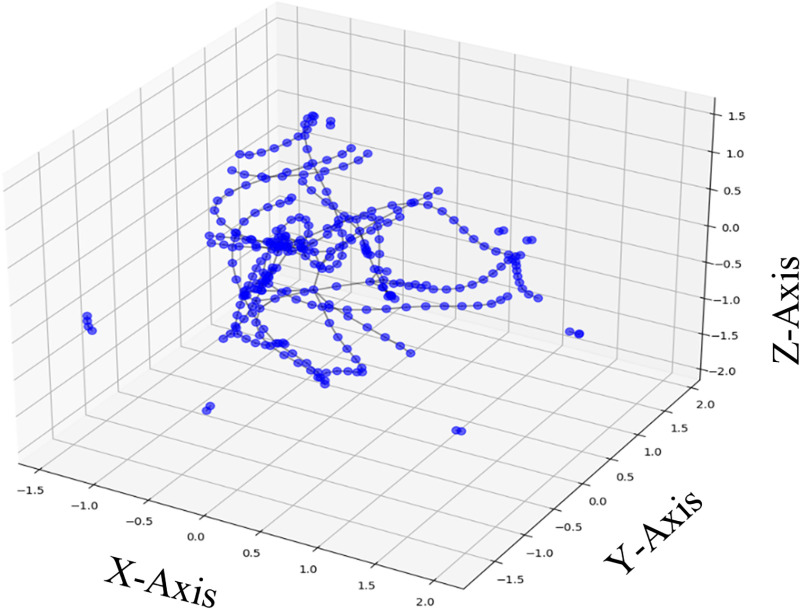
The spatial network of Sensors.

In this study, we incorporate both spatial and temporal features to enhance the model’s ability to predict traffic flow accurately. The spatial features capture real-time, dynamic relationships among traffic sensors, while the temporal segments, including hourly, daily, and weekly intervals, capture recurring patterns over different time scales for each sensor. Fig 2 illustrates that the sensors are represented as blue dots, with lines indicating dynamically updated connections based on real-time traffic conditions. This approach allows the model to capture dynamic spatial relationships that adjust with traffic flow variations, effectively reflecting immediate changes in the sensor network structure.

In addition to adjacent sensors, the model also incorporates non-adjacent sensor relationships to account for long-range dependencies. Adjacent sensors provide immediate spatial correlations, which are essential for localized traffic predictions. However, non-adjacent sensors, even those that are physically distant, can exhibit similar patterns over time due to recurring traffic trends, such as periodic congestion or shared routes.

The proximity and density of sensors are also significant factors in the model. In urban environments, sensor distribution is uneven; certain areas, such as intersections, have densely connected sensors, while others, such as less frequented roads, have sparse connections. This variability is evident in the sensor layout, where clusters of connected sensors contrast with isolated points. By capturing these proximity and density variations, the model can accurately represent areas of high traffic flow as well as isolated sections with unique traffic patterns.

**Fig 3:**
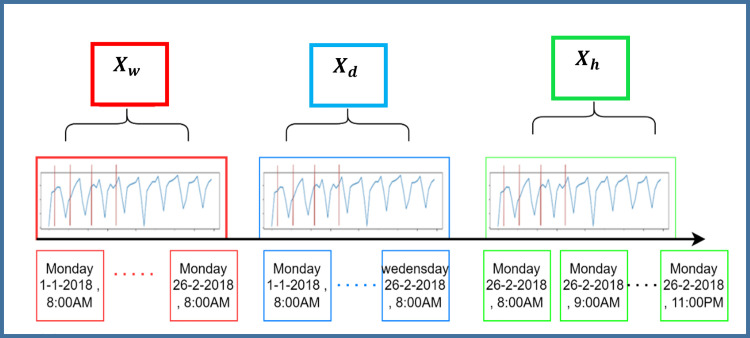
Recurrent patterns in time series data.


The input data X is organized to capture different patterns over time, as shown in Fig 3. The data is divided into three main segments: hourly, daily, and weekly, each tailored to identify specific recurring trends. The first segment labelled ${\text{X}}_{\text{h}}$ and highlighted in green, focuses on the most recent hour leading up to the current moment. This segment utilizes data from the hour immediately before the target prediction period on February 26, 2018, from 08:00–09:00 AM. It is represented mathematically as:

$${\text{X}}_{\text{h}}=\left({\text{X}}_{\text{t}-{\text{T}}_{\text{h}}+\text{1}},{\text{X}}_{\text{t}-{\text{T}}_{\text{h}}+\text{2}},\ldots ,{\text{X}}_{\text{t}}\right)$$ (5)

The weekly segment (green) focuses on the recurrent pattern at 8 AM from different week, the daily segment (blue) captures recurring traffic at 8 AM in different days, and the hourly segment (red) identifies the traffic pattern of the recent hours. The second segment, shown in blue and labelled ${\text{X}}_{\text{d}}$, captures daily patterns by examining data from the same timeframe on previous days, such as January 1 and 2, 2018. This helps the model learn from past days to predict traffic flow for the current day. It is expressed as:


$$\begin{array}{c} {\text{X}}_{\text{d}}=({\text{X}}_{\text{t }-\left({\text{T}}_{\text{d}}/\text{T}\right)*\text{q}+\text{1}},\ldots ,{\text{X}}_{\text{t }-\left({\text{T}}_{\text{d}}/\text{T}\right)*\text{q}+\text{T}},{\text{X}}_{\text{t }-(\text{T}/\text{T}-\text{1})*\text{q}+\text{1}},\ldots ,\\ {\text{X}}_{\text{t}-\left({\text{T}}_{\text{d}}/\text{T}-\text{1}\right)*\text{q}+\text{T}},\ldots ,{\text{X}}_{\text{t}-\text{1}*\text{q}+\text{1}},\ldots ,{\text{X}}_{\text{t}-\text{1}*\text{q}+\text{T}}) \end{array}$$ (6)

The third segment, indicated by the red lines and labelled ${\text{X}}_{\text{w}}$, captures weekly recurring patterns by using data from the same hours but from previous weeks, specifically from January 1 to January 26, 2018. This is formulated as:


$$\begin{array}{c} {\text{X}}_{\text{w}}=({\text{X}}_{\text{t }-\text{7}*\left({\text{T}}_{\text{w}}/\text{T}\right)*\text{q}+\text{1}},\ldots ,{\text{X}}_{\text{t}-\text{7}*\left({\text{T}}_{\text{w}}/\text{T}\right)*\text{q}+\text{T}},{\text{X}}_{\text{t }-\text{7}*\left({\text{T}}_{\text{w}}/\text{T}-\text{1}\right)*\text{q}+\text{1}},\ldots ,\\ {\text{X}}_{\text{t }-\text{7}*\left({\text{T}}_{\text{w}}/\text{T}-\text{1}\right)*\text{q}+\text{T}},\ldots ,{\text{X}}_{\text{t }-\text{7}*\text{1}*\text{q}+\text{1}},\ldots ,{\text{X}}_{\text{t }-\text{7}*\text{1}*\text{q}+\text{T}}) \end{array}$$ (7)

By combining these segments, the model can recognize both short-term fluctuations and longer-term patterns in traffic, leading to more accurate predictions. This approach is essential for understanding and predicting traffic dynamics, providing a solid foundation for the model’s effectiveness.

### 3.4. Integrated spatiotemporal feature alignment

**Fig 4:**
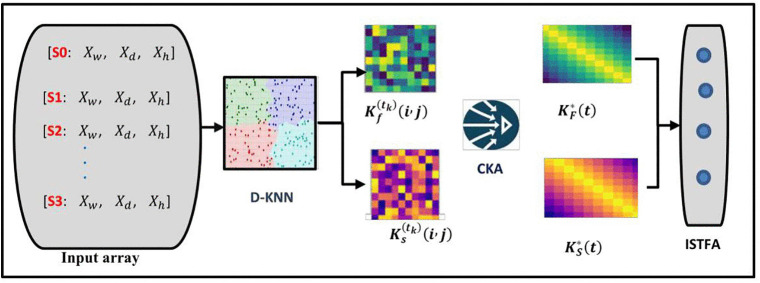
Integrated spatiotemporal feature extraction alignment.

The Integrated Spatiotemporal Feature Alignment (ISTFA) module is designed to address a key limitation in existing traffic prediction models, namely the inability to jointly and dynamically integrate spatial proximity with fine-grained temporal and feature-level similarity. As shown in Fig 4, ISTFA operates on multi-resolution traffic data, including hourly, daily, and weekly observations, and constructs an adaptive representation that evolves with traffic conditions. The importance of ISTFA lies in its ability to align spatial and temporal relationships in real time, enabling the model to distinguish between sensors that are geographically close but behaviorally different, and those that are spatially distant yet temporally correlated. By combining Dynamic K-Nearest Neighbours (D-KNN) with Centered Kernel Alignment (CKA), ISTFA produces an interpretable and adaptive graph structure that enhances responsiveness to abrupt traffic changes and improves the robustness of downstream graph convolutional learning.

### 3.5. Dynamic neighbourhood matrix construction

Dynamic KNN is used to build an adaptive neighbourhood structure that reflects the evolving nature of traffic flow. Unlike static KNN, where neighbours remain fixed, the neighbourhood in this approach is updated at every time step. This is achieved by recalculating similarity scores, adjusting the similarity threshold dynamically, selecting the most relevant neighbouring sensors, and weighting their contributions according to their importance for prediction.

In a real traffic network such as PeMSD4, which contains 307 loop detectors, this means that the neighbours of a given sensor at 07:00 AM during morning congestion may differ from those at 02:00 AM when traffic is light, even though the physical road network itself does not change.

At each time step t, the similarity between traffic sensors is measured using the Pearson correlation coefficient across multiple temporal resolutions, including hourly $X_{h}$, daily $X_{d}$, and weekly $X_{w}$ patterns. This allows the model to capture both short-term fluctuations and long-term trends in traffic behaviour.

The similarity between sensor i and sensor j within the time window $\left[t_{a},t_{b}\right]$ is computed as:


$$\begin{array}{c} C_{ij}(t)=\frac{\sum_{t=t_{a}}^{t_{b}}(X_{d[i]t}-{\bar{X}}_{i})(X_{d[j]t}-{\bar{X}}_{j})}{\sqrt{\sum_{t=t_{a}}^{t_{b}}(X_{d[i]t}-{\bar{X}}_{i}{)}^{\text{2}}}\sqrt{\sum_{t=t_{a}}^{t_{b}}(X_{d[j]t}-{\bar{X}}_{j}{)}^{\text{2}}}} \end{array}$$ (8)

Here, $C_{ij}(t)$ represents the similarity score between sensors i and j at time t, while ${\bar{X}}_{i}$ and ${\bar{X}}_{j}$ denote their mean traffic values over the selected interval. For example, two sensors located on different road segments may still show a high similarity score if congestion builds up and dissipates at the same time during peak hours.

Once similarity scores are computed, a binary selection rule is applied to determine whether two sensors should be considered neighbours:


$$\begin{array}{c} S_{ij}(t)=\left\{ \begin{array}{cc} \text{1}, & \text{if}|C_{ij}(t)|>\theta (t)\\ \text{0}, & \text{otherwise} \end{array}\right. \end{array}$$ (9)

The threshold θ(t)is not fixed. Instead, it is updated dynamically based on the distribution of similarity values at time t:


$$\begin{array}{c} \theta (t)=\left\{ \begin{array}{cc} |C_{ij}(t){|}_{\frac{n+\text{1}}{\text{2}}}, & \text{if}n\text{is}\mathrm{odd}\\ \frac{|C_{ij}(t){|}_{\frac{n}{\text{2}}}+|C_{ij}(t){|}_{\frac{n}{\text{2}}+\text{1}}}{\text{2}}, & \text{if}n\text{is}\mathrm{even} \end{array}\right. \end{array}$$ (10)

Here, n denotes the total number of sensors. In practice, this mechanism raises the threshold during periods of widespread congestion, keeping only the strongest relationships, and lowers it during off-peak hours, allowing a broader set of neighbours to be considered.

For each sensor i at time $t_{k}$, the top K neighbours are selected and combined across different temporal patterns:


$$\begin{array}{c} D_{i,j,t_{k}}=w_{d}D(X_{i,d,t_{k}},X_{j,d,t_{k}})+w_{h}D(X_{i,h,t_{k}},X_{j,h,t_{k}})+w_{w}D(X_{i,w,t_{k}},X_{j,w,t_{k}}) \end{array}$$ (11)

The weights $w_{d}$, $w_{h}$, and $w_{w}$ control the relative importance of daily, hourly, and weekly information. For instance, during weekday rush hours, short-term hourly patterns often dominate, whereas weekly trends become more informative during weekends.

To fully describe sensor relationships, two complementary kernel matrices are constructed from the dynamic neighbourhood information.

Then, the spatial kernel matrix captures proximity-based relationships derived mainly from daily traffic behaviour:


$$\begin{array}{c} K_{s}=\{K_{s}(t),K_{s}(t+\text{1}),\ldots ,K_{s}(T)\} \end{array}$$ (12)

Each matrix $K_{s}(t_{k})\in R^{n\times n}$ is defined as:


$$\begin{array}{c} K_{s}(t_{k})(i,j)=w_{d}\cdot D(X_{i,d,t_{k}},X_{j,d,t_{k}}) \end{array}$$ (13)

Lower values in this matrix indicate stronger spatial affinity, reflecting sensors that exhibit similar daily traffic patterns.

In parallel, a feature similarity kernel is constructed to capture temporal behaviour beyond spatial proximity:


$$\begin{array}{c} K_{f}=\{K_{f}(t),K_{f}(t+\text{1}),\ldots ,K_{f}(T)\} \end{array}$$ (14)

with entries computed as:


$$\begin{array}{c} K_{f}(t_{k})(i,j)=w_{h}\cdot D(X_{i,h,t_{k}},X_{j,h,t_{k}})+w_{w}\cdot D(X_{i,w,t_{k}},X_{j,w,t_{k}}) \end{array}$$ (15)

This kernel highlights sensors that behave similarly over short and long-time horizons, even if they are geographically distant.

### 3.6. Kernel alignment using CKA

To integrate spatial and feature-based relationships, Centered Kernel Alignment (CKA) is applied. The alignment score between the two kernels is given by:


$$\begin{array}{c} U(K_{s}(t),K_{f}(t))=\frac{\langle K_{s}(t),K_{f}(t)\rangle_{F}}{∥K_{s}(t){∥}_{F}\cdot ∥K_{f}(t){∥}_{F}} \end{array}$$ (16)

A higher alignment score indicates stronger consistency between spatial proximity and temporal behaviour, guiding the model toward a more meaningful kernel combination.

The aligned spatial and feature kernels are obtained as:


$$\begin{array}{c} \overset{*}{\underset{S}{K}}(t)=\sum_{p=\text{1}}^{P_{S}}\overset{p}{\underset{S}{\alpha }}(t)\overset{p}{\underset{S}{K}}(t) \end{array}$$ (17)

$$\begin{array}{c} \overset{*}{\underset{F}{K}}(t)=\sum_{q=\text{1}}^{P_{F}}\overset{q}{\underset{F}{\alpha }}(t)\overset{q}{\underset{F}{K}}(t) \end{array}$$ (18)

Finally, the Integrated Spatiotemporal Feature Alignment (ISTFA) matrix is formed as:


$$\begin{array}{c} K_{H}(t)=[\begin{array}{cc} \overset{*}{\underset{S}{K}}(t) & A\\ A^{T} & \overset{*}{\underset{F}{K}}(t) \end{array}]\in R^{n\times n} \end{array}$$ (19)

This matrix jointly represents spatial proximity, temporal behaviour, and their interactions. It serves as the adjacency structure for the GCN layers, enabling effective information propagation and improving traffic flow prediction accuracy. The overall procedure for constructing the ISTFA similarity kernel, including dynamic neighbourhood selection, threshold adaptation, kernel construction, and CKA-based alignment, is formally summarized in Algorithm 1.

(Input array represents Traffic data (daily, hourly, weekly) is processed through Dynamic KNN to capture spatial relationships, generating spatial adjacency ($\overset{\left(t_{k}\right)}{\underset{s}{K}}$) and feature similarity ($\overset{\left(t_{k}\right)}{\underset{f}{K}}$) matrices which are by CKA to produce the arrays ($\overset{*}{\underset{F}{K}}(t)$ and $\overset{*}{\underset{S}{K}}(t)$.Then, they are integrated into the ISTFA array, which serves as input to the GCNN

**Algorithm1.** **ISTFA**

**Input:**

• D: hist*orical dataset {x₁, x₂, …, x₍ₘ ₋ ₁₎}*

• *X₀: feature vector of the target sensor at time t*

• *P: sensor position matrix*

• *k: number of neighbours*

• *ε: small constant*

• *θ_min, θ_max: minimum and maximum similarity thresholds*

• *M₀: number of missing sensors*

• *M: total number of sensors*

• *γ: threshold scaling parameter*

• *βₛ, β𝒻:* spatial and feature fusion weights

***Output:***

• *K_ISTFA(t): ISTFA similarity kernel matrix at time t*

***Begin***

 *m ← |D|;*

  *distances ← NULL;*

  *X_e ← NULL;*

  *X_d ← NULL;*

  *for i ← 1 to m do*

  *Compute distᵢ between xᵢ and X₀;*

   *Add (distᵢ, xᵢ) to distances;*

 *end for*

  *Sort distances in ascending order;*

  *X_e ← first k elements of distances;*

  *D_s ← (M₀ / M) × 100;*

  *θ ← θ_min + (θ_max − θ_min) × (1 − γ × D_s / 100);*

  *for each (distᵢ, xᵢ) in distances do*

  *if distᵢ < θ then*

   *Add xᵢ to X_d;*

  *end if*

  *end for*

  *Compute weights wᵢ ← 1 / (distᵢ + ε);*

  *Normalize all weights.*

  *X_c(t) ← weighted combination of X_e and X_d;*

  *Construct spatial kernel Kₛ(t) using P and X_c(t);*

  *Construct feature kernel K𝒻(t) using X_c(t);*

  *Apply CKA to align Kₛ(t) and K𝒻(t);*

 Obtain optimized kernels Kₛ*(t) and K𝒻*(t);

  K_ISTFA(t) ← [Kₛ*(t) A Aᵀ  K𝒻*(t)];

  *Return K_ISTFA(t);*

***End***

### 3.7. Spatial learning

This lays the groundwork for the next critical phase—feeding the enriched array ${\text{K}}_{\text{H }}(\text{t})$ into a GCN for intricate traffic flow learning. The array ${\text{K}}_{\text{H }}(\text{t})$ is primed to serve as the initial feature matrix ${\text{H}}^{\left(\text{0}\right)}$ for our GCN:


$${\text{H}}^{\left(\text{0}\right)}={\text{K}}_{\text{H }}(\text{t})$$ (20)

This initial representation ${\text{H}}^{\left(\text{0}\right)}$ encompasses the comprehensive sensor relationships at time t and is pivotal for the GCN to discern and exploit the underlying structure within the traffic network. The first layer of the GCN receives ${\text{H}}^{\left(\text{0}\right)}$ and performs the following operation:


$${\text{H}}^{\left(\text{1}\right)}=\sigma \left({\hat{\text{D}}}^{-\frac{\text{1}}{\text{2}}}\hat{\text{A}}{\hat{\text{D}}}^{-\frac{\text{1}}{\text{2}}}{\text{H}}^{\left(\text{0}\right)}{\text{W}}^{\left(\text{0}\right)}\right)$$ (21)

**Fig 5:**
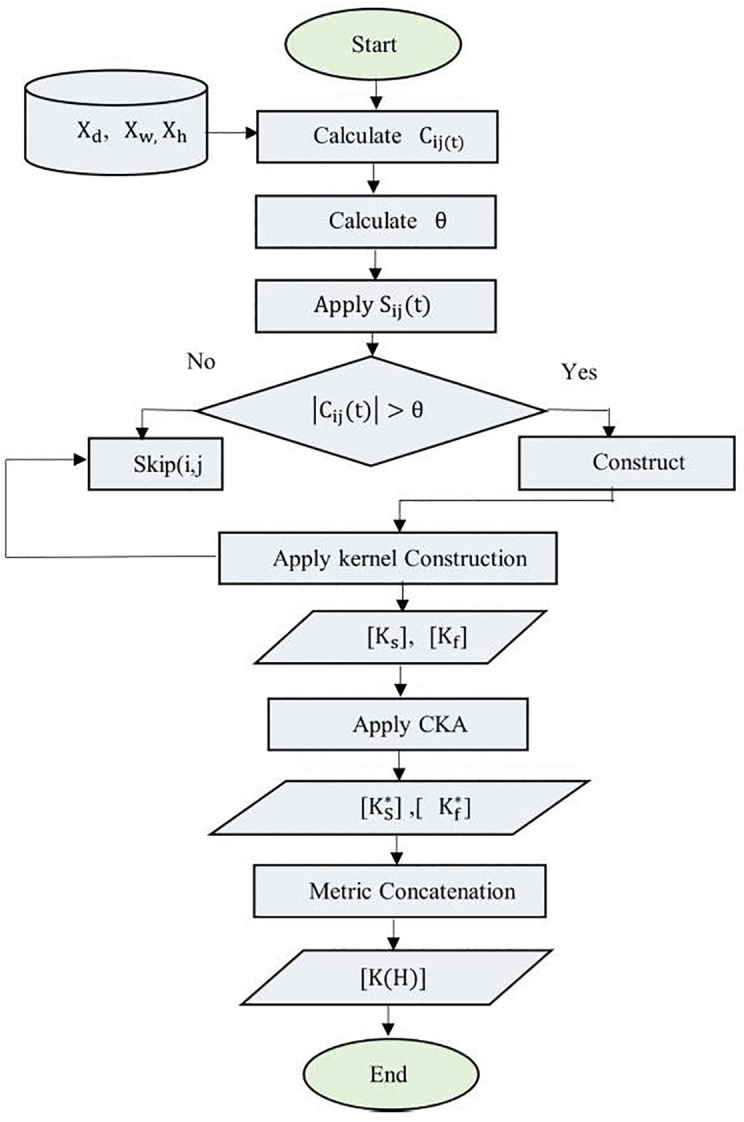
Flowchart of the ISTFA array.

Here, ${\text{H}}^{\left(\text{1}\right)}$ signifies the nodes’ feature representations after the first convolutional layer, σ represents a non-linear activation function(ReLU), to introduce non-linearity into the model, $\hat{\text{A}}$ is the adjacency matrix of the graph with self-connections, facilitating the inclusion of a node’s features in the update process, $\hat{\text{D}}$ is the degree matrix corresponding to inclusion of a node’s features in the update process, $\hat{\text{D}}$ is the degree matrix corresponding to $\hat{\text{A}}$ ensuring that the feature aggregation is normalized and ${\text{W}}^{\left(\text{0}\right)}$ is the weight matrix for the first layer, which is subject to optimization during the training phase. Fig 5 presents the flowchart of the ISTFA array.

**Fig 6:**
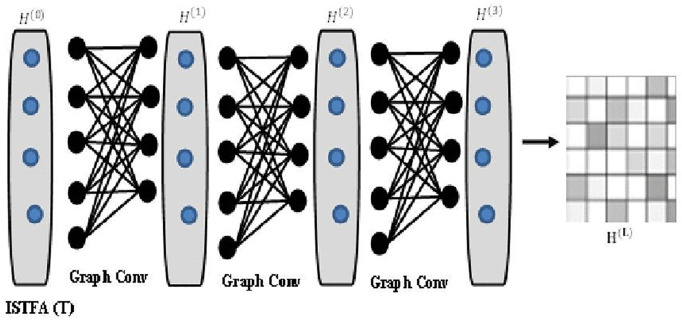
Graph Convolution Network Architecture [36].


The output ${\text{H}}^{\left(\text{1}\right)}$then acts as the input for the subsequent layer. The final output of the GCN ${\text{H}}^{\left(\text{L}\right)}$after layers can be aligned with the traffic flow labels through a prediction layer. Fig 6 shows the architecture of the input and output matrices of GCN in the spatial learning module.

$${\text{H}}^{\left(\text{L}\right)}=\sigma \left({\hat{\text{D}}}^{-\frac{\text{1}}{\text{2}}}\hat{\text{A}}{\hat{\text{D}}}^{-\frac{\text{1}}{\text{2}}}{\text{H}}^{\left(\text{L}-\text{1}\right)}{\text{W}}^{\left(\text{L}-\text{1}\right)}\right)$$ (22)

### 3.8. Temporal leaning

**Fig 7:**
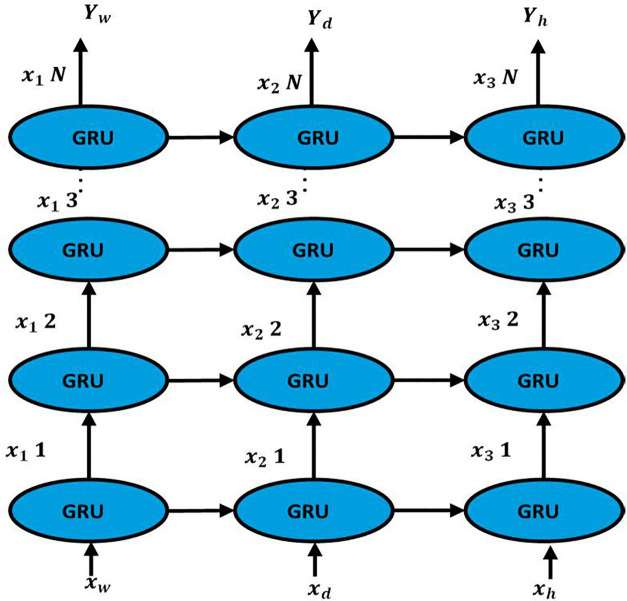
Gated Recurrent Unit Network Architecture.

The temporal module, depicted in Fig 7 is designed to capture, and model the sequential dependencies across different time scales—specifically, weekly, daily, and hourly patterns. This module integrates these temporal dependencies using a series of GRU layers arranged in a multi-layered structure, with each layer corresponding to a specific temporal pattern. The input to the temporal module consists of three distinct sequences: ${\text{X}}_{\text{w},\text{i}}$, ${\text{X}}_{\text{d},\text{i}}$, and ${\text{X}}_{\text{h},\text{i}}$, representing weekly, daily, and hourly data at the i-th sensor, respectively. Each GRU cell receives inputs corresponding to these temporal patterns. For example, ${\text{X}}_{\text{w},\text{1}}$, ${\text{X}}_{\text{d},\text{1}}$, and ${\text{X}}_{\text{h},\text{1}}$, represent the features for the first sensor, while ${\text{X}}_{\text{w},\text{2}}$, ${\text{X}}_{\text{d},\text{2}}$, and ${\text{X}}_{\text{h},\text{2}}$ correspond to the second sensor, continuing in this manner for all N sensors. The GRU cells process these inputs at each time step to generate hidden states ${\text{h}}_{\text{w},\text{i}}$, ${\text{h}}_{\text{d},\text{i}}$, and ${\text{h}}_{\text{h},\text{i}}$, which capture the temporal dependencies within each time scale. The operation of each GRU cell is governed by the following equations:

1. Update gate:

$${\text{z}}^{\left(\text{t}\right)}=\sigma \left({\text{W}}_{\text{z}}.{[\text{h}}^{\left(\text{t}-\text{1}\right)},{\text{X}}_{\text{t}}]\right)$$ (23)

Where, ${\text{z}}^{\left(\text{t}\right)}$ determines the extent to which the previous hidden state ${\text{h}}^{\left(\text{t}-\text{1}\right)}$ will contribute to the current hidden state. ${\text{W}}_{\text{z}}$ represents the weight matrix for the update gate, and σis the sigmoid activation function.

2. Reset Gate ${\text{r}}^{\left(\text{t}\right)}$:

$${\text{r}}^{\left(\text{t}\right)}=\sigma \left({\text{W}}_{\text{r}}.{[\text{h}}^{\left(\text{t}-\text{1}\right)},{\text{X}}_{\text{t}}]\right)$$ (24)

The reset gate ${\text{r}}^{\left(\text{t}\right)}$ decides how much of the previous hidden state should be forgotten. ${\text{W}}_{\text{r}}$ is the weight matrix associated with the reset gate.

3. Candidate Hidden State$\overset{―}{\text{h}}(\text{t})$:

$$\overset{―}{\text{h}}(\text{t})=\text{tanh}\left({\text{W}}_{\text{h}}.{[{\text{r}}^{\left(\text{t}\right)}*\text{h}}^{\left(\text{t}-\text{1}\right)},{\text{X}}_{\text{t}}]\right)$$ (25)

The candidate hidden state $\overset{―}{\text{h}}(\text{t})$ represents the new content that could be added to the current hidden state, modulated by the reset gate. ${\text{W}}_{\text{h}}$ is the weight matrix for the candidate hidden state, and tanh denotes the hyperbolic tangent activation function.

4. Final Hidden State ${\text{h}}^{\left(\text{t}\right)}$:

$${\text{h}}^{\left(\text{t}\right)}=\left(\text{1}-{\text{z}}^{\left(\text{t}\right)}\right){*\text{h}}^{\left(\text{t}-\text{1}\right)}+{\text{z}}^{\left(\text{t}\right)}*\overset{―}{\text{h}}(\text{t})$$ (26)

The final hidden state ${\text{h}}^{\left(\text{t}\right)}$ is a combination of the previous hidden state ${\text{h}}^{\left(\text{t}-\text{1}\right)}$ and the candidate hidden state $\overset{―}{\text{h}}(\text{t})$, weighted by the update gate.

The hidden states are updated according to these GRU equations, where the update gate ${\text{z}}^{\left(\text{t}\right)}$, reset gate ${\text{r}}^{\left(\text{t}\right)}$, and new hidden state ${\text{h}}^{\left(\text{t}\right)}$ are computed to maintain the temporal continuity of the sequences. The architecture allows for horizontal connections between the GRU cells within the same row, which propagate temporal information forward across the time sequence. Additionally, vertical connections between different rows, representing different temporal scales, enable the integration of multi-scale temporal features, ensuring that each temporal level informs the others. The final hidden states from each temporal scale, denoted as ${\text{Y}}_{\text{w},\text{i}}$, ${\text{Y}}_{\text{d},\text{i}}$, and ${\text{Y}}_{\text{h},\text{i}}$ for weekly, daily, and hourly outputs respectively, are then used to make the final predictions. These outputs are effectively combined to generate a comprehensive prediction that encapsulates information from all three temporal scales. This temporal module is a critical component of the model, integrating multi-scale temporal data to enhance prediction accuracy. The structured configuration of the GRU cells ensures that both short-term and long-term dependencies are captured, providing predictions that are informed by comprehensive temporal insights.

### 3.9. Prediction module

**Fig 8:**
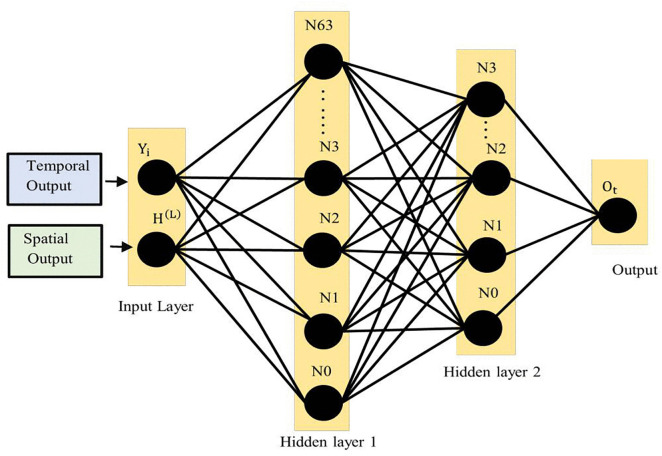
Fully connected layer for prediction module.


The Prediction Module, shown in Fig 8 combines the features learned from the spatial relationships captured by the GCN and the time-based patterns modelled by the GRU network to produce a final traffic flow prediction. The inputs to these FCL are the outputs from the temporal module, labelled as ${\text{Y}}_{\text{i}}$, and the outputs from the spatial module, labeled as ${\text{H}}^{\left(\text{L}\right)}$. These outputs represent the time-based features and the spatial relationships between different traffic sensors. These input vectors are combined into a single feature vector, which is then fed into the first FCL. The fully connected structure includes two hidden layers. The first hidden layer receives the combined feature vector as its input. Each node in this layer is represented as ${\text{N}}_{\text{j}}$, where j= 1, 2, 63). The process in this layer can be described by the equation:

$${\text{Z}}_{\text{1}}=\sigma \left({\text{W}}_{\text{1}}.[{\text{Y}}_{\text{i}},{\text{H}}^{\left(\text{1}\right)}+{\text{b}}_{\text{1}}]\right)$$ (27)

where ${\text{Z}}_{\text{1}}$ is the output of the first hidden layer, ${\text{W}}_{\text{1}}$ is the weight matrix, ${\text{b}}_{\text{1}}$ is the bias vector, and σ is the activation function, a ReLU function. ReLU is chosen as the activation function due to its ability to introduce non-linearity into the model while maintaining computational efficiency. Non-linearity is crucial for capturing complex traffic flow patterns that a linear model would miss. ReLU is defined as:


σ(x)=max(0,x) (28)

This simple yet powerful function outputs the input directly if it is positive and outputs zero otherwise. Its use helps mitigate the vanishing gradient problem, common in deep networks, thereby ensuring faster convergence and more stable training. The output from the first hidden layer, ${\text{Z}}_{\text{1}}$, is then fed into the second hidden layer, which has nodes represented as ${\text{N}}_{\text{k}}$, where k = 1, 2,  .  .  .,  31. The operation in this layer is given by:


$${\text{Z}}_{\text{2}}=\sigma \left({\text{W}}_{\text{2}}.[{\text{Y}}_{\text{i}},{\text{H}}^{\left(\text{2}\right)}+{\text{b}}_{\text{2}}]\right)$$ (29)

Where ${\text{Z}}_{\text{2}}$ is the output of the second hidden layer, ${\text{W}}_{\text{2}}$ is the weight matrix, and ${\text{b}}_{\text{2}}$ is the bias vector. Finally, the second hidden layer’s output, ${\text{Z}}_{\text{2}}$, is passed into the output layer, which generates the predicted traffic flow value, represented by ${\text{O}}_{\text{t}}$.This final prediction is calculated using the equation:


$${\text{O}}_{\text{t}}={\text{W}}_{\text{3}}.{\text{Z}}_{\text{2}}+{\text{b}}_{\text{3}}$$ (30)

Where ${\text{W}}_{\text{3}}$ is the weight vector connecting the second hidden layer to the output, and ${\text{b}}_{\text{3}}$ is the bias term. The use of ReLU activation in the hidden layers helps the model effectively learn and capture complex patterns in the data. The final output ${\text{O}}_{\text{t}}$ is then compared with the actual traffic flow to measure the prediction error, using MSE. This ensures that the fully connected layers effectively translate the spatial and temporal features learned by the model into accurate and reliable traffic flow predictions.

## 4. Experiments

**Fig 9:**
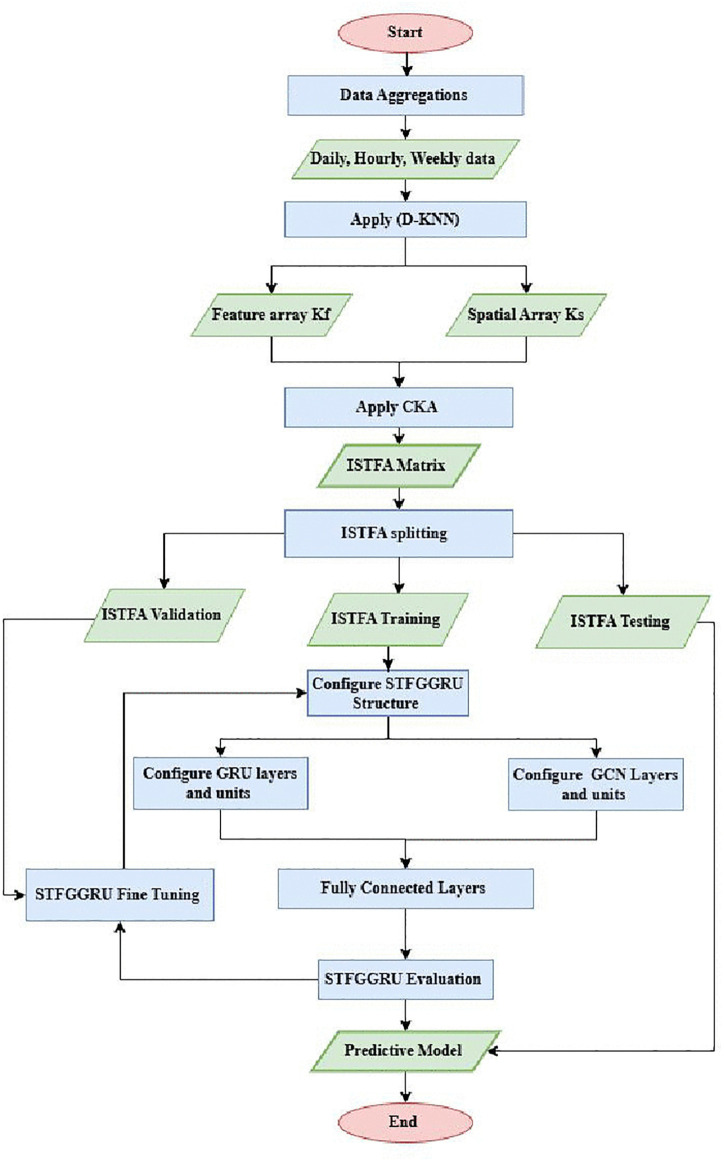
Flowchart of STF-GGRU implementation.

The implementation of the STF-GGRU model, as illustrated in Fig 9 begins with data aggregation into daily, hourly, and weekly intervals to capture temporal patterns. The D-KNN method extracts feature arrays (Kf) and spatial arrays (Ks), which are aligned using CKA to generate the ISTFA matrix, encapsulating spatiotemporal dependencies. The ISTFA matrix is then split into validation, training, and testing subsets for hyperparameter tuning, model training, and evaluation. The STF-GGRU structure is configured with GRU layers for temporal dependencies and GCN layers for spatial relationships, followed by fully connected layers for feature integration. The model undergoes fine-tuning and evaluation to produce the final predictive model for traffic flow forecasting. Each step of this process will be explained in detail in the subsequent sections.

## 5. Datasets

This research employs the PeMSD4 and PeMSD8 datasets, which are well-established benchmarks for traffic flow prediction. Tables 3, 4 showcase samples from these datasets. The PeMSD4 dataset comprises traffic data collected from 307 sensors between January 1, 2018, and February 28, 2018. In contrast, the PeMSD8 dataset includes data from 170 sensors recorded from July 1, 2016, to August 31, 2016. Both datasets feature records from multiple sensors, capturing essential metrics such as traffic flow, occupancy, and speed at various timestamps. For example, in the PeMSD4 dataset, Sensor 0 recorded a flow of 62 vehicles on “2018-01-01T00:00:00Z,” with an occupancy rate of 0.0074 and a speed of 67.3 km/h. Similarly, in the PeMSD8 dataset, Sensor 0 recorded a flow of 133 vehicles on “2016-07-01T00:00:00Z,” with an occupancy rate of 0.0637 and a speed of 65.8 km/h.

**Table 3:** PeMSD4 traffic flow prediction dataset.

Dyna ID	Type	Time	Sensor ID	Traffic flow	Traffic occupancy	Traffic speed
**0**	State	2018-01-01T00:00:00Z	0	62.0	0.0077	67.9
**1**	State	2018-01-01T00:05:00Z	0	61.0	0.0074	67.3
**2**	State	2018-01-01T00:10:00Z	0	71.0	0.0093	68.4
**3**	State	2018-01-01T00:15:00Z	0	86.0	0.0112	67.8
**4**	state	2018-01-01T00:20:00Z	0	103.0	0.0144	67.4
. . .	. . .	. . .	. . .	. . .	. . .	. . .
**5216541**	state	2018-02-28T23:45:00Z	306	57.0	0.0166	67.8
**5216542**	state	2018-02-28T23:50:00Z	306	43.0	0.0118	69.1
**5216543**	state	2018-02-28T23:55:00Z	306	40.0	0.0107	69.7

**Table 4:** PeMSD8 traffic flow prediction dataset.

Dyna ID	Type	Time	Sensor ID	Traffic flow	Traffic occupancy	Traffic speed
**0**	state	2016-07-01T00:00:00Z	0	133.0	0.0603	65.8
**1**	state	2016-07-01T00:05:00Z	0	114.0	0.0532	66.9
**2**	state	2016-07-01T00:10:00Z	0	140.0	0.0622	66.8
**3**	state	2016-07-01T00:15:00Z	0	106.0	0.0452	68.9
. . .	. . .	. . .	. . .	. . .	. . .	. . .
**3035516**	state	2016-08-31T23:40:00z	169	2.0	0.0007	65.4
**3035517**	state	2016-08-31T23:45:00Z	169	3.0	0.0014	65.3
**3035518**	state	2016-08-31T23:50:00Z	169	2.0	0.001	65.2
**3035519**	state	2016-08-31T23:55:00Z	169	6.0	0.0026	65.2

## 6. Evaluation of metrics and baseline models

To evaluate the performance of the proposed model, three key metrics have been employed which are Root Mean Squared Error (RMSE) and Mean Absolute Error (MAE) and Mean Absolute Percentage Error (MAPE). These metrics assess the accuracy of the predicted traffic flow, denoted as ${\hat{\text{Y}}}_{\text{t}}$= {${\hat{\text{y}}}_{\text{1}}$, ${\hat{\text{y}}}_{\text{2}},\ldots ,{\hat{\text{y}}}_{\text{N}}$}, compared to the actual observed traffic flow, ${\text{Y}}_{\text{t}}$= {${\text{y}}_{\text{1}}$, ${\text{y}}_{\text{2}},\ldots ,{\text{y}}_{\text{N}}$}, where N represents the number of stations in the traffic network.

RMSE measures the standard deviation of the prediction errors, giving greater weight to larger errors. Lower RMSE values indicate higher prediction accuracy, especially when large prediction errors are minimized. It is calculated as:


$$\text{RMSE}=\sqrt{\frac{\text{1}}{\text{n}}\sum_{\text{i}=\text{1}}^{\text{n}}\;{\left({\hat{\text{y}}}^{\text{i}}-{\text{y}}^{\text{i}}\right)}^{\text{2}}}$$ (31)

MAE provides the average magnitude of the prediction errors, offering a straightforward measure of accuracy by averaging absolute differences between predicted and actual values. It is determined by:


$$MAPE=\frac{\text{1}}{n}\sum_{i=\text{1}}^{n}\;\frac{\left|{\hat{y}}^{i}-y^{i}\right|}{y^{i}}\times \text{100}\%$$ (32)

$$\text{MAE}=\frac{\text{1}}{\text{n}}\sum_{\text{i}=\text{1}}^{\text{n}}\;\left|{\hat{\text{y}}}^{\text{i}}-{\text{y}}^{\text{i}}\right|$$ (33)

MAPE expresses error as a percentage, highlighting the model’s accuracy in relative terms. It’s particularly useful for understanding prediction performance across different scales in traffic flow. It is determined by:

Both RMSE and MAE offer insights into the differences between predicted and actual traffic flows, with lower values indicating higher prediction accuracy. The proposed STFGGRU model for long-term traffic flow prediction is compared against the following baseline methods:

1. Autoregressive Integrated Moving Average (ARIMA) [37]: This model treats the temporal data sequence as a stochastic sequence and utilizes autocorrelation analysis to forecast future values based on the historical time series data.
2. Gated Recurrent Unit (GRU) [38]:This model used GRU models for urban traffic flow prediction. By integrating traffic data, episodic events, and weather information, the research aims to improve the accuracy of traffic congestion forecasts.
3. Long Short-Term Memory (LSTM) [39]: This model addresses the issue of gradient vanishing using a “gate” mechanism, making it a popular choice for time series prediction. It takes the traffic flow from the previous time step as input to predict the flow at the next time step.
4. K- Nearest Neighbour LSTM (KNN-LSTM) [40]: This model based on KNN, and two layers of LSTM can capture the spatial dependence based on the most related neighbouring stations and mine the variability of the traffic flow.
5. Spatiotemporal Graph Convolutional Network (STGCN) [41]: This model based on fixed Laplacian matrix for spatial temporal data.
6. Multi-Component Spatiotemporal GCN (MSTGCN) [42]: This is a spatiotemporal graph convolution model incorporating a spatiotemporal attention mechanism. It utilizes the road network structure to model station relationships, forming the foundation for graph convolution. It has demonstrated remarkable accuracy in predicting road traffic flow.
7. Attention-based Periodic-Temporal Neural Network (APTN) [33]:This model uses spatiotemporal attention to capture complex dependencies by incorporating encoder and temporal attention mechanisms. Its ability to enable inter-node interactions while preventing overfitting makes it effective for traffic prediction challenges.

### 6.1. Experiment setup

The PeMSD4 and PeMSD8 datasets were used to evaluate the STF-GGRU model, split into training, validation, and test sets with a 7:1.5:1.5 ratio, and 5-fold cross-validation ensured robust results. Input features (traffic flow, occupancy, speed) were normalized to [0, 1], and data was aggregated into hourly, daily, and weekly segments to capture multi-scale temporal patterns. In terms of architecture, the model includes GRU layers and a two-layer GCN with 64 units each. Key hyperparameters were optimized: a learning rate of 0.001, batch size of 64, dropout rate of 0.3, and up to 100 training epochs with early stopping and a learning rate scheduler reducing the rate by 0.1 if validation loss plateaued for 10 epochs. To evaluate model sensitivity, increasing GCN layers from 1 to 3 reduced RMSE by 8% on PeMSD4, and 64 GRU units were found to balance efficiency and accuracy. Additionally, a dynamic KNN threshold of 0.7 in ISTFA improved MAE by 5%. To ensure robust experimentation, experiments were conducted on Google Collab Pro (Intel Xeon, 13GB RAM, NVIDIA Tesla T4 GPU) using TensorFlow, PyTorch, and Scikit-learn. Metrics such as RMSE, MAE, and MAPE benchmarked STF-GGRU against baseline models, including ARIMA, GRU, LSTM, KNN-LSTM, STGCN, MSTGCN, and APTN. Finally, paired t-tests (0.05 significance) confirmed the importance of hyperparameter optimization for achieving state-of-the-art performance in traffic flow.

## 7. Results

### 7.1. Comparison with other baseline methods

**Fig 10:**
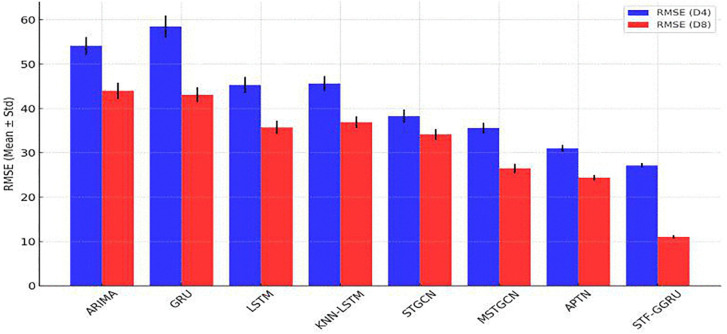
RMSE of STFFGRU and Baselines.

The STF-GGRU model was evaluated against baseline models using RMSE, MAE, and MAPE metrics on the PeMSD4 and PeMSD8 datasets, as summarized in Table 5, 6. The results were obtained by averaging the outcomes over 5 independent runs with different random seeds to ensure robustness, and the standard deviations are reported to reflect performance variability. These comparisons demonstrate STF-GGRU’s ability to significantly outperform traditional and neural network-based models due to optimized parameter choices and its advanced spatiotemporal modelling capabilities. In particular, the dynamic KNN threshold in the ISTFA module was set to 0.7, ensuring effective spatiotemporal feature alignment. Additionally, the use of two GCN layers enabled the model to capture intricate spatial dependencies across sensors, while the inclusion of 64 GRU units balanced the ability to model long-term temporal patterns with computational efficiency. These parameter optimizations resulted in a significant reduction in RMSE values, achieving 27.18 ± 0.85 for PeMSD4 and 11.01 ± 0.50 for PeMSD8. In contrast, the GRU model recorded an RMSE of 58.5 ± 2.5 on PeMSD4, and ARIMA showed higher RMSE values of 54.14 ± 2.00 on PeMSD4 and 44 ± 1.80 on PeMSD8 as shown in Fig 10.

**Table 5:** Evaluation metrics of Benchmarks and STF-GGRU on PeMSD4.

Model	RMSE (D4)	RMSE (D8)	MAE (D4)	MAE (D8)	MAPE (D4)	MAPE (D8)
**ARIMA**	54.14 ± 2	44 ± 1.8	36.76 ± 1.5	29.52 ± 1.4	25.3 ± 1.2	18.6 ± 1.0
**GRU**	58.5 ± 2.5	43.1 ± 1.7	32.11 ± 1.2	24.5 ± 1.1	23.5 ± 1.1	15.9 ± 0.9
**LSTM**	45.31 ± 1.8	35.76 ± 1.5	29.23 ± 1.1	23.6 ± 1.0	21.2 ± 1.0	15.43 ± 0.8
**KNN-LSTM**	45.62 ± 1.7	36.91 ± 1.3	29.45 ± 1.0	23.19 ± 0.9	21.1 ± 0.9	15.2 ± 0.7
**STGCN**	38.3 ± 1.5	34.17 ± 1.2	24.15 ± 0.8	21.72 ± 0.8	20.4 ± 0.8	14.64 ± 0.6
**MSTGCN**	35.6 ± 1.2	26.47 ± 1.1	26.06 ± 0.7	17.46 ± 0.6	19.86 ± 0.7	14 ± 0.5
**APTN**	31 ± 0.8	24.4 ± 0.6	22.78 ± 0.5	15.62 ± 0.4	11.9 ± 0.4	8.31 ± 0.3
**STF-GGRU**	27.18 ± 0.5	11.01 ± 0.4	16.2 ± 0.3	10.75 ± 0.3	9.67 ± 0.3	6.97 ± 0.2

**Table 6:** Evaluation metrics of Benchmarks and STF-GGRU on PeMSD8.

Model	RMSE (D4)	RMSE (D8)	MAE (D4)	MAE (D8)	MAPE (D4)	MAPE (D8)
**ARIMA**	54.14 ± 2	44 ± 1.8	36.76 ± 1.5	29.52 ± 1.4	25.3 ± 1.2	18.6 ± 1.0
**GRU**	58.5 ± 2.5	43.1 ± 1.7	32.11 ± 1.2	24.5 ± 1.1	23.5 ± 1.1	15.9 ± 0.9
**LSTM**	45.31 ± 1.8	35.76 ± 1.5	29.23 ± 1.1	23.6 ± 1.0	21.2 ± 1.0	15.43 ± 0.8
**KNN-LSTM**	45.62 ± 1.7	36.91 ± 1.3	29.45 ± 1.0	23.19 ± 0.9	21.1 ± 0.9	15.2 ± 0.7
**STGCN**	38.3 ± 1.5	34.17 ± 1.2	24.15 ± 0.8	21.72 ± 0.8	20.4 ± 0.8	14.64 ± 0.6
**MSTGCN**	35.6 ± 1.2	26.47 ± 1.1	26.06 ± 0.7	17.46 ± 0.6	19.86 ± 0.7	14 ± 0.5
**APTN**	31 ± 0.8	24.4 ± 0.6	22.78 ± 0.5	15.62 ± 0.4	11.9 ± 0.4	8.31 ± 0.3
**STF-GGRU**	27.18 ± 0.5	11.01 ± 0.4	16.2 ± 0.3	10.75 ± 0.3	9.67 ± 0.3	6.97 ± 0.2

**Fig 11:**
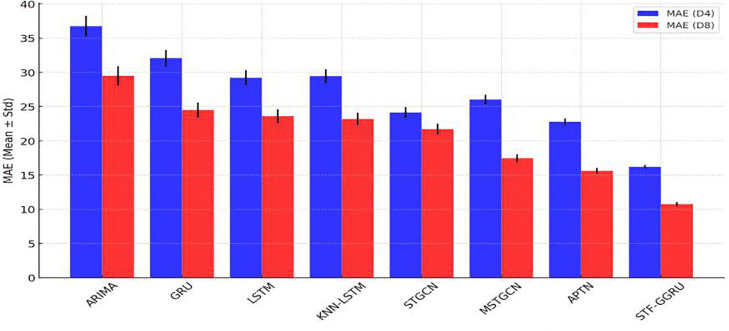
MAE of STFFGRU and Baselines.

**Fig 12:**
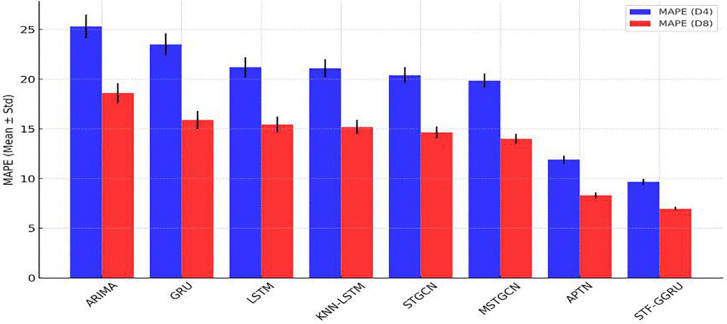
MAPE of STFFGRU and Baseline.

Further illustrating its effectiveness, MAE results confirmed STF-GGRU’s superior performance. The model achieved the lowest MAE values of 16.2 ± 0.60 for PeMSD4 and 10.75 ± 0.40 for PeMSD8. By comparison, ARIMA, GRU, and KNN-LSTM exhibited much higher MAE values with greater variability. This performance was supported by a carefully tuned learning rate of 0.001 and a dropout rate of 0.3, which enhanced convergence stability and mitigated overfitting as shown in Fig 11. Additionally, where error bars represent the standard deviation across 5 independent runs. This highlights the model’s consistent performance and confirms minimal overlap with competing method. The MAPE measures the average percentage error between predicted and actual values, with lower values indicating higher accuracy. Fig 12 compares the MAPE of various models, highlighting the STF-GGRU model’s superior performance due to optimized spatiotemporal feature extraction. All results are averaged over five independent runs, with standard deviations included to ensure reliability and illustrate performance variability. For the PeMSD4 dataset, traditional models like ARIMA (25.3 ± 1.2%) and GRU (23.5 ± 1.1%) exhibited higher MAPE values, while LSTM (21.2 ± 1.0%) and KNN-LSTM (21.1 ± 0.9%) showed moderate improvements. Advanced spatiotemporal models, STGCN (20.4 ± 0.8%) and MSTGCN (19.86 ± 0.7%), further reduced errors. STF-GGRU achieved the lowest MAPE of 9.67 ± 0.3%, attributed to parameters like a dynamic KNN threshold of 0.7 and two GCN layers for capturing intricate spatial dependencies. In the less complex PeMSD8 dataset, all models performed better. ARIMA (18.6 ± 1.0%), GRU (15.9 ± 0.9%), and LSTM (15.43 ± 0.8%) showed steady improvements, while KNN-LSTM (15.2 ± 0.7%) and STGCN (14.64 ± 0.6%) demonstrated further accuracy gains. MSTGCN reduced errors to 14 ± 0.5%, but STF-GGRU achieved the lowest MAPE of 6.97 ± 0.2%. The learning rate of 0.001 and a dropout rate of 0.3 played critical roles in preventing overfitting and ensuring convergence, validating the robustness of STF-GGRU across datasets. Overall, these results highlight the importance of strategic parameter selection, optimization, and robustness in evaluation. The STF-GGRU model’s performance on PeMSD4 and PeMSD8 demonstrates its ability to deliver state-of-the-art accuracy in traffic flow prediction while maintaining stability across multiple runs, surpassing other benchmark models.

### 7.2. Comparison of STF-GGRU and other baseline methods under data variability

Model Performance Variability Analysis includes two examples that assess how well the model can adjust to different kinds of conditions. Using data collected from a variety of sensors, Case 1 investigates its spatial precision. Case 2 examines how well the model consistently predicts different traffic volumes. The collective analysis assesses the model’s reliability and practicality in predicting traffic conditions.

### 7.3. Case1: Scalability of STF-GGRU at different sensors

**Fig 13:**
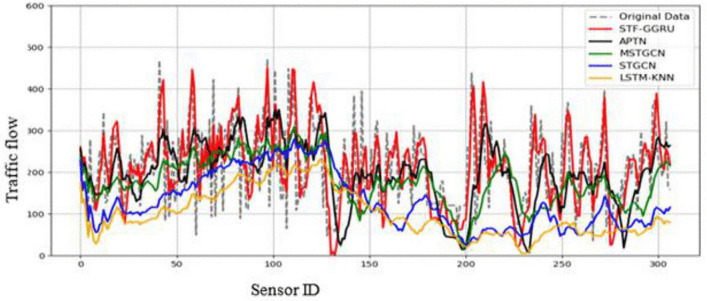
Prediction for STF-GGRU and benchmark on PeMSD4.

**Fig 14:**
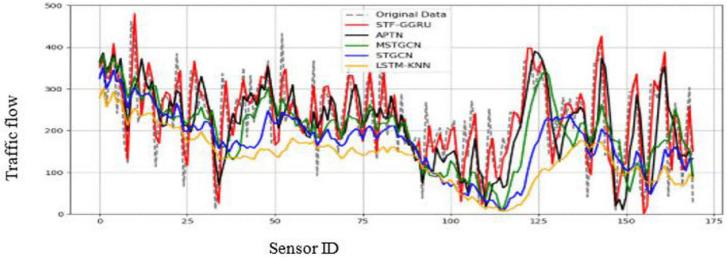
Prediction for STF-GGRU and benchmark on PeMSD8.

**Fig 15:**
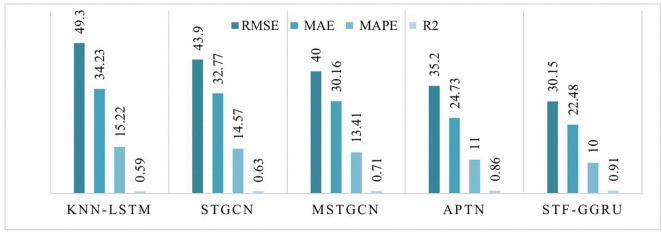
Performance for STF-GGRU and Benchmark on all sensors On PeMSD8.

**Fig 16:**
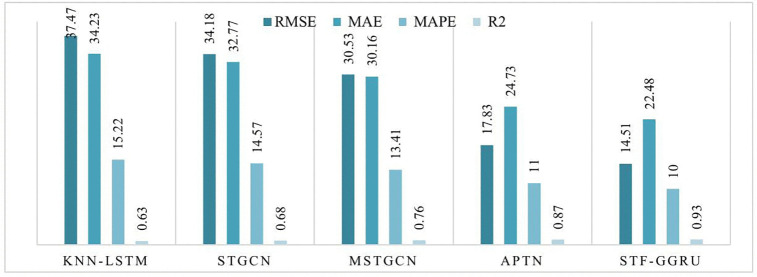
Performance for STF-GGRU and benchmark on all sensors On PeMSD8.

This case study examines the scalability and predictive performance of the proposed STF-GGRU model in comparison with several benchmark methods across all sensors in the PeMSD4 and PeMSD8 datasets. The strong performance of STF-GGRU is mainly attributed to the effective integration of dynamic KNN-based graph construction, stacked GCN layers, GRU-based temporal modeling, and the ISTFA module. Careful parameter tuning, including optimization of the dynamic KNN threshold, selection of two GCN layers, and a GRU hidden size of 64, played an important role in improving model adaptability and prediction accuracy. As shown in Fig 13, STF-GGRU closely follows the ground-truth traffic patterns on PeMSD4 and clearly outperforms the baseline models, demonstrating its ability to capture complex spatiotemporal relationships. Similarly, Fig 14 illustrates stable and reliable performance on the PeMSD8 dataset despite the smaller number of sensors, highlighting the robustness of the proposed model. The quantitative results presented in Figs 15, 16 further confirm the superiority of STF-GGRU. On PeMSD4, the model achieves the lowest RMSE of 30.15, MAE of 22.48, and MAPE of 10, along with the highest coefficient of determination with an R² value of 0.91, outperforming APTN and other competing approaches. These performance trends are consistently maintained on PeMSD8, where STF-GGRU again records the lowest prediction errors and the highest R² of 0.93, demonstrating strong generalization across datasets with different network sizes.

In terms of computational complexity, the STF-GGRU model combines GCNs, GRUs, and the ISTFA module to capture complex spatiotemporal dependencies. Although this integrated design leads to state-of-the-art prediction accuracy, it also results in increased computational requirements. The GCN component has a complexity of $O(L_{GCN}\cdot |V|\cdot d^{\text{2}})$, where the quadratic dependence on the feature dimension dbecomes computationally expensive, especially in dense graph structures. The GRU component operates with a complexity of $O(L_{GRU}\cdot T\cdot n\cdot m)$, which remains relatively efficient but increases with the length of the temporal sequence. The ISTFA module introduces a complexity of $O(|V{|}^{\text{2}}\cdot d)$and represents the main source of computational overhead, particularly for large-scale sensor networks. Overall, the model complexity can be expressed as $O(L_{GCN}\cdot |V|\cdot d^{\text{2}})+O(L_{GRU}\cdot T\cdot n\cdot m)+O(|V{|}^{\text{2}}\cdot d)$, highlighting the trade-off between high predictive performance and computational cost.

Despite these computational demands, STF-GGRU remains feasible for medium-sized datasets such as PeMSD4 and PeMSD8 when deployed on modern GPU platforms. However, real-time and large-scale deployment scenarios may benefit from further algorithmic optimizations, including sensor clustering strategies and sparse matrix computations, to reduce computational and memory overhead. This scalability is further validated by the experimental results presented in Table 7, where the number of nodes varied from 50 to 300. Across this range, prediction accuracy remains stable, with only marginal increases observed in MAE, RMSE, and MAPE. At the same time, training time, GPU memory consumption, and inference latency increase gradually and remain within practical limits. These results confirm that STF-GGRU scales effectively while maintaining a strong balance between accuracy and efficiency. In summary, STF-GGRU not only achieves state-of-the-art predictive performance but also demonstrates robust scalability, making it well suited for real-world deployment in large-scale urban traffic management systems.

**Table 7:** Scalability performance of the proposed model.

Number of Nodes	MAE	RMSE	MAPE (%)	Train Time (s/epoch)	GPU Memory (GB)	Inference Time (ms)
50	2.03	3.70	5.0	9.5	5.5	18
100	2.09	3.78	5.3	17.2	7.3	23
200	2.14	3.86	5.6	30.4	9.8	31
300	2.20	3.94	6.0	45.8	12.0	42

### 7.4. Performance of the model prediction at different traffic volumes

**Fig 17:**
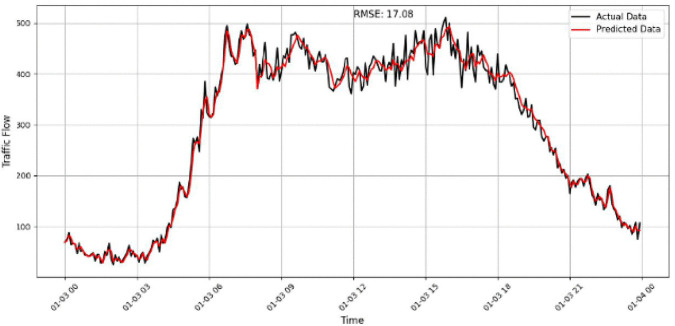
Traffic flow prediction results for Sensor 16 on the PeMSD4.

**Fig 18:**
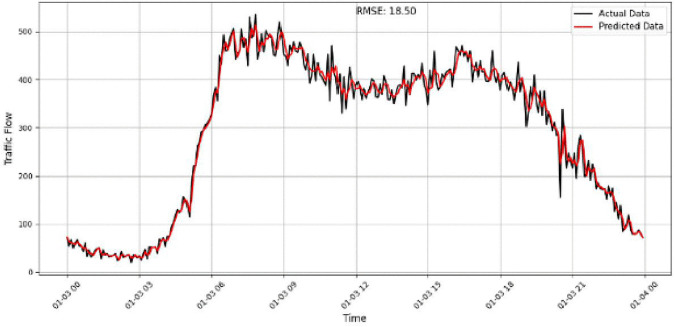
Traffic flow prediction results for Sensor 250 on the PeMSD4.

**Fig 19:**
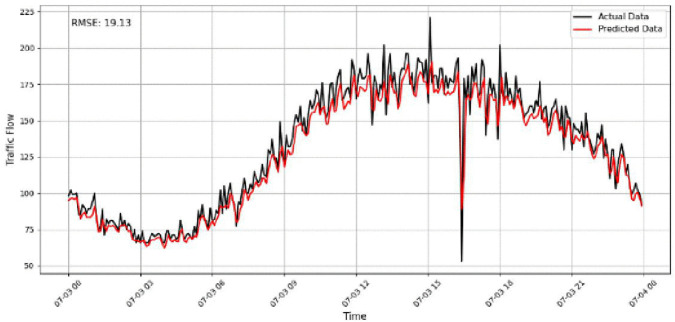
Traffic flow prediction results for Sensor 14 on the PeMSD8.

**Fig 20:**
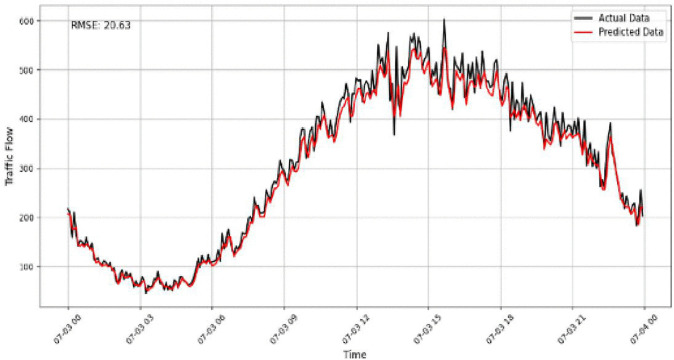
Traffic flow prediction results for Sensor 105 on the PeMSD8.

A focused analysis of our traffic prediction model’s performance across various sensor volumes and times uses RMSE as the key metric to assess accuracy. By comparing actual and predicted traffic data, we evaluate the model’s ability to capture traffic patterns, particularly during rush hours and transitional periods. The comparison between the predicted and actual traffic flow is illustrated in Figs 17–20 for four representative sensors from the PeMSD4 and PeMSD8 datasets. Specifically, Fig 17 presents the prediction results for Sensor 16 on PeMSD4, while Fig 18 shows the results for Sensor 250 on PeMSD4. The corresponding RMSE values of 17.8 and 15.79 indicate that the proposed model accurately captures the underlying traffic trends for both sensors. In addition, Fig 19 illustrates the prediction performance for Sensor 14 on PeMSD8, which achieves the lowest RMSE value of 13, demonstrating superior accuracy on this dataset. Finally, Fig 20 depicts the results for Sensor 105 on PeMSD8, further confirming the model’s ability to closely follow real traffic dynamics across different sensors and network scales.

**Fig 21:**
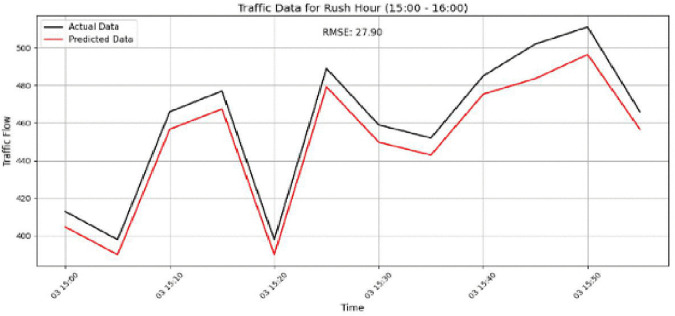
Prediction result for Sensor 250 on the PeMSD4 dataset during the 15:00–16:00 rush hour.

**Fig 22:**
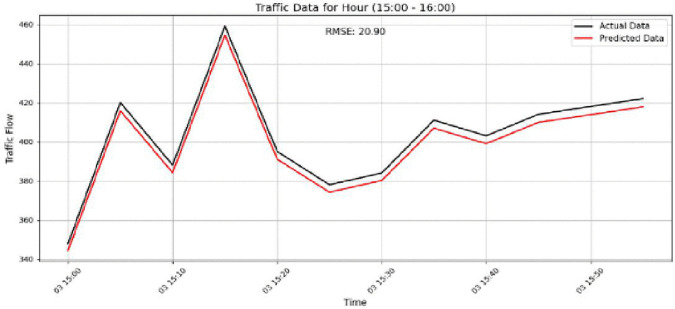
Prediction results for Sensor 160 on the PeMSD4 dataset during the rush hour period.

**Fig 23:**
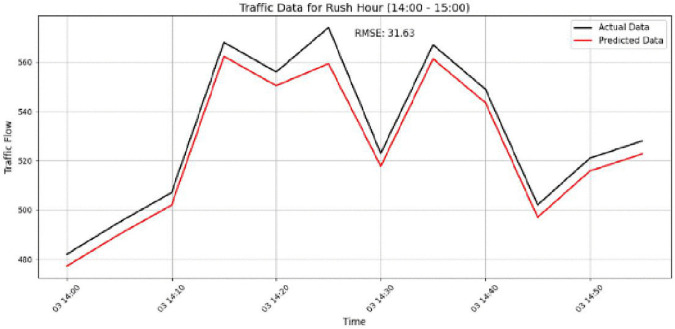
Prediction results for Sensor 14 on the PeMSD8 dataset during the rush hour.

**Fig 24:**
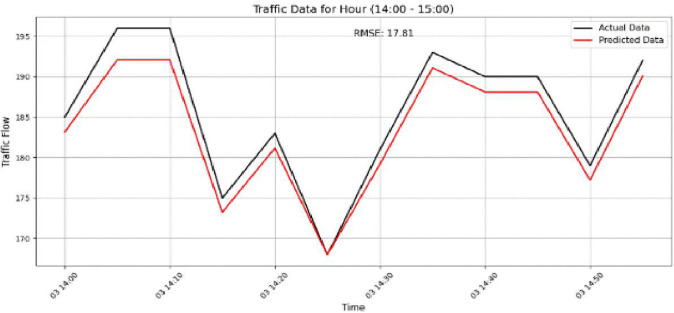
Prediction result for Sensor 105 on the PeMSD8 dataset during the 14:00–15:00 period.

The model performance is further examined in Figs 21–24. Fig 21 shows the prediction results for Sensor 250 on the PeMSD4 dataset during the 15:00–16:00 period, where an RMSE of 27.96 indicates the increased difficulty of prediction under highly variable traffic conditions. Fig 22 presents the results for Sensor 160 on PeMSD4 during the rush hour, achieving better accuracy with an RMSE of 20.90, as the predicted values closely follow the actual traffic patterns. For the PeMSD8 dataset, Fig 23 illustrates the results for Sensor 14 during the rush hour, which records the lowest RMSE of 18.43, highlighting the model’s strong performance during peak traffic periods. In addition, Fig 24 shows the prediction results for Sensor 105 on PeMSD8 during the 14:00–15:00 period, with an RMSE of 17.81, demonstrating reliable performance even during transitional traffic conditions. Overall, these results suggest that the model performs more effectively during rush-hour periods than during non-rush or transitional intervals. Although prediction accuracy varies slightly across sensors, the consistently reasonable RMSE values across all cases confirm that the model provides reliable and accurate traffic flow predictions.

## 8. The ablation analysis of STF-GGRU

**Fig 25:**
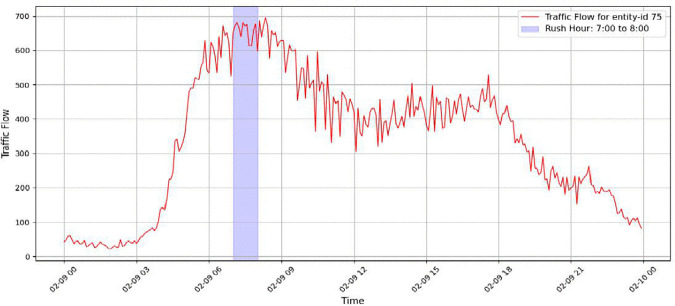
The highest traffic data PeMSD4 on 2-9-2018 (rush hour).

The STF-GGRU model is designed to capture traffic flow patterns by jointly modelling temporal evolution, spatial relationships, and adaptive feature importance. To examine how each component contributes to prediction accuracy, an ablation study is conducted using data from Sensor 75 in the PeMSD4 dataset. This sensor recorded the highest traffic volume on February 9, 2018, particularly during the morning rush hour shown in Fig 25. The traffic profile on this day is highly dynamic, showing a rapid rise in flow, pronounced peaks, and noticeable fluctuations. These characteristics make it a suitable case for evaluating the model under realistic and challenging conditions.

**Fig 26:**
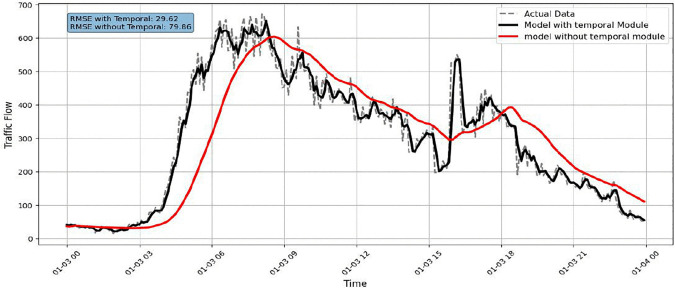
Model performance without Temporal Module.

As illustrated in Fig 25, traffic flow increases sharply during the rush-hour period and then gradually decreases. Several short-term variations are also observed throughout the day. Accurately capturing this behaviour requires a model that can respond quickly to changes over time. This requirement is clearly reflected in Fig 26, where the temporal module is removed. Without temporal modelling, the predicted traffic curve becomes overly smooth and lags the actual data. The model struggles to follow sudden increases and sharp drops in traffic, particularly during peak congestion. As a result, prediction accuracy deteriorates significantly. The RMSE increases to approximately 79.86, compared to 29.62 when the temporal module is included. This large gap confirms that temporal modelling is the most influential factor in achieving accurate traffic predictions.

**Fig 27:**
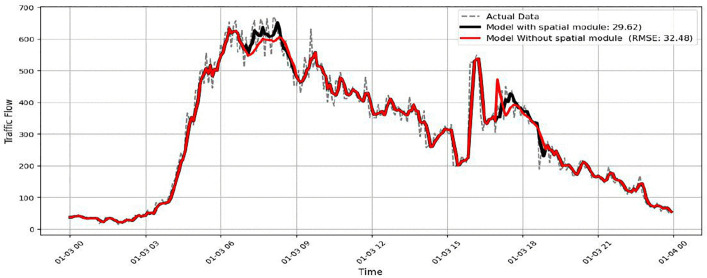
Model performance without Spatial Module.

The importance of spatial relationships is examined in Fig 27 by removing the spatial module. In this case, the model continues to follow the overall temporal pattern. However, noticeable deviations from the actual traffic flow appear during transition phases, such as the buildup and release of congestion. These differences suggest that information from neighbouring sensors helps refine local predictions. Without spatial modelling, the RMSE increases to about 32.48. This result indicates that spatial modelling provides a meaningful improvement, although its impact is smaller than that of temporal modelling and adaptive feature extraction.

**Fig 28:**
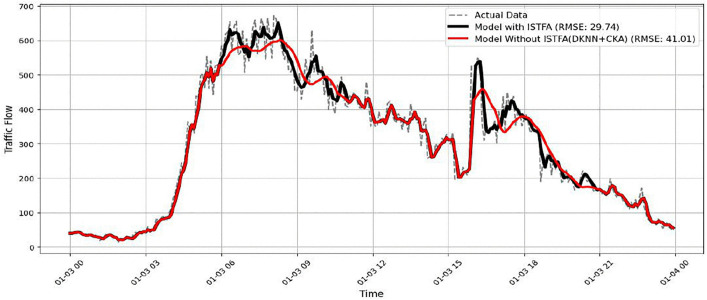
Model performance without ISTFA.

In addition to temporal modelling, STF-GGRU relies on the ISTFA module to adaptively highlight the most relevant spatiotemporal features. When this module is removed, as shown in Fig 28, the predicted traffic flow becomes less responsive to rapid changes. Peak values are underestimated, and fluctuations are smoothed out, especially during busy periods. Although the general trend is still captured, the model fails to reflect the true intensity of congestion. This limitation leads to an RMSE of approximately 41.01, compared to 29.74 for the complete model. These results show that adaptive feature extraction plays a key role in handling complex traffic behaviour.

**Fig 29:**
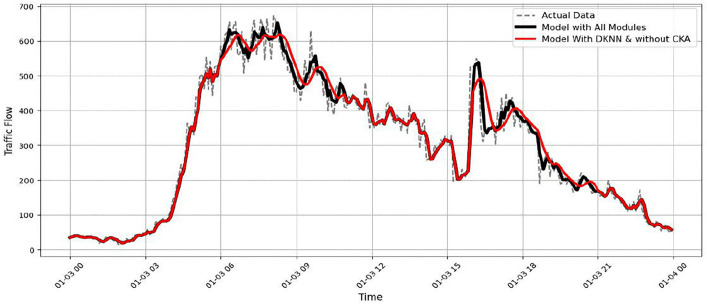
Model performance without CKA Module.

**Fig 30:**
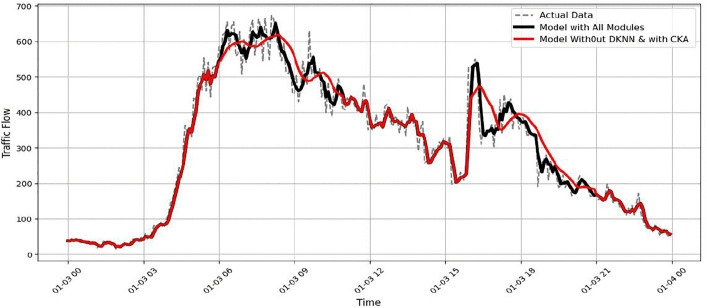
Model performance without DKNN Module.

Further insight is gained by analysing the individual elements within the ISTFA module. Fig 29 shows the effect of removing the CKA alignment while keeping the D-KNN mechanism. The predictions remain generally accurate but display slight inconsistencies. Alignment with peak values is also reduced. This behaviour results in an RMSE of approximately 33.9. The result suggests that CKA helps stabilize feature representations and improve consistency, even if its impact is relatively subtle. Similarly, Fig 30 illustrates the effect of removing the D-KNN mechanism. Without dynamic neighbour selection, the model becomes less sensitive to sudden traffic changes, particularly during peak periods. This leads to an RMSE of approximately 35.4. These findings indicate that D-KNN contributes to adaptability under varying traffic densities.

Overall, the ablation results show that the temporal module has the strongest influence on prediction accuracy. It is followed by the ISTFA module, which enhances the model’s ability to adapt to complex and rapidly changing traffic patterns. The spatial module further improves prediction quality by capturing interactions between sensors. The D-KNN and CKA components provide additional gains through adaptability and representation stability. Together, these components allow STF-GGRU to produce accurate and reliable traffic flow predictions under challenging real-world conditions.

## 9. Conclusion and future work

In this paper, we introduced STF-GGRU, a novel traffic flow prediction framework designed to address the limitations of existing models in handling complex, nonlinear, and dynamic urban traffic patterns. The key innovation of our approach is the Integrated Spatiotemporal Feature Alignment (ISTFA) module, which uniquely combines Dynamic K-Nearest Neighbor (D-KNN) and Centered Kernel Alignment (CKA) dynamically integrate both spatial proximity and feature-based similarities into a unified graph representation. Through extensive experiments on large-scale real-world datasets (METR-LA and PEMS-BAY), STF-GGRU demonstrated superior predictive accuracy compared to state-of-the-art baseline models across multiple short-term and long-term horizons. Our additional scalability analysis further validated the model’s robustness and computational efficiency under varying network sizes and sensor densities, highlighting its practical viability for deployment in large-scale intelligent transportation systems and real-time urban mobility management. The proposed STF-GGRU model can be practically implemented within modern urban traffic management systems by integrating it into either centralized traffic control centers or distributed edge computing infrastructures, such as roadside processing units or local traffic servers. Real-time traffic flow data collected from existing sensing networks, including loop detectors, traffic cameras, GPS-equipped vehicles, and connected vehicle systems, can be streamed directly to the model for continuous prediction without requiring fundamental changes to current infrastructure. The adaptive nature of the ISTFA module allows STF-GGRU to process dynamic traffic changes promptly and provide timely flow forecasts that can inform signal control, congestion mitigation strategies, and route guidance services. Furthermore, computational cost analysis shows that the model maintains moderate training times, reasonable GPU memory usage, and fast inference speeds (ranging from 18 to 42 milliseconds), ensuring its practicality for real-time deployment. The model can also be periodically updated using newly collected data to sustain accuracy over time. These features collectively enable STF-GGRU to be seamlessly integrated into existing smart city platforms and intelligent transportation infrastructures, supporting more efficient, adaptive, and data-driven urban mobility management. In contrast to prior works that primarily focus on either static spatial relationships or simplified temporal dependencies, STF-GGRU offers a comprehensive and adaptive framework capable of modeling intricate spatiotemporal correlations. This original contribution opens new avenues for advanced traffic prediction research and provides a solid foundation for developing intelligent, responsive, and scalable traffic management solutions in smart cities. Future work will focus on integrating external factors such as weather conditions, major events, and multi-modal transportation data to further enhance prediction accuracy and system resilience. Additionally, exploring edge and fog computing deployment architectures will be pursued to support distributed, real-time traffic control applications.
